# Supplementary material for: HPV-YAP1 oncogenic alliance drives malignant transformation of fallopian tube epithelial cells
Source: EMBO Rep. 2024 Sep 13;25(10):26. doi: 10.1038/s44319-024-00233-3 (PMC11467260; doi:10.1038/s44319-024-00233-3)
Supplement: Supplementary file 1 — Appendix [file 44319_2024_233_MOESM1_ESM.pdf]

APPENDIX

Table of content

Content	*Page number*
Table of content	1
Appendix Figure S1	2
Appendix Figure S2	3
Appendix Figure S3	4
Appendix Figure S4	5
Appendix Figure S5	6
Appendix Figure S6	7
Appendix Figure S7	8
Appendix Figure S8	9
Appendix Figure S9	10
Appendix Figure S10	11
Appendix table S1	12

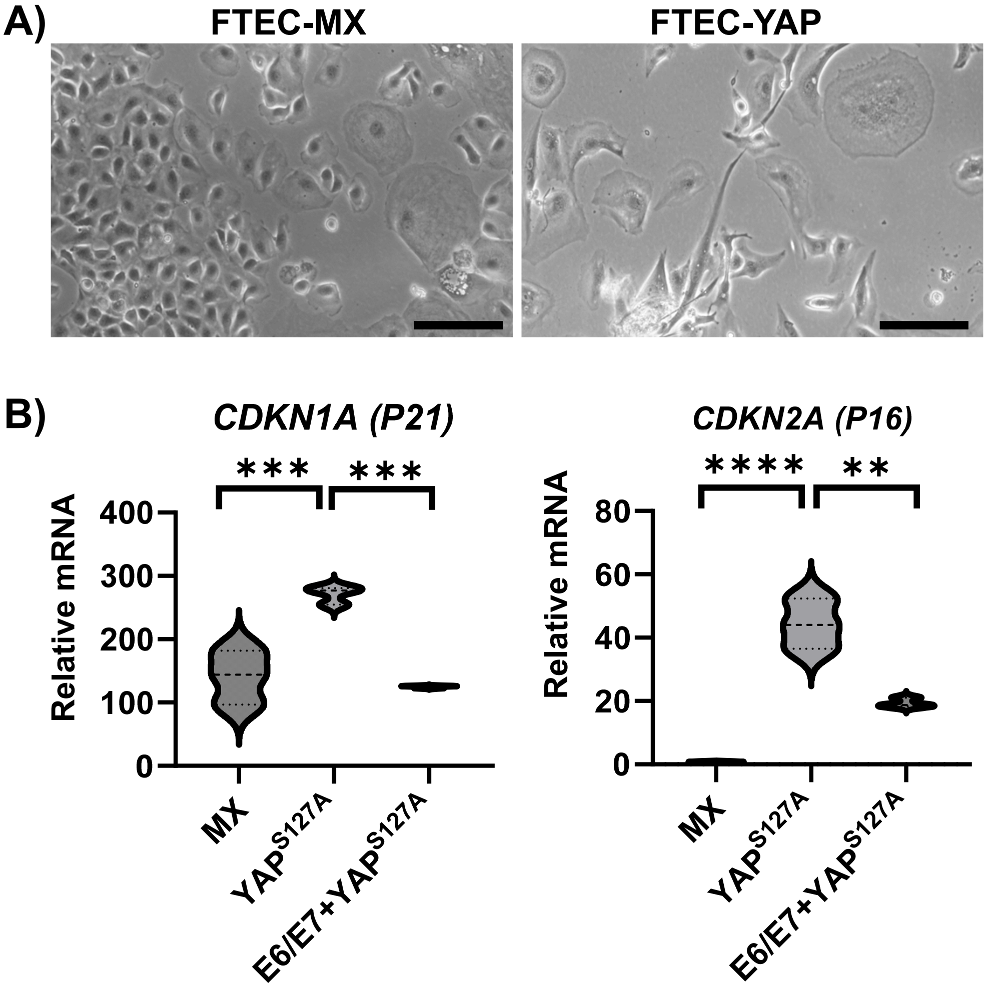

Appendix Figure S1. Ectopic expression of YAP protein in promoted cellular senescence in the cultured primary FTECs.

A) Representative images showing that overexpression of YAP1 in cultured primary FTECs induced cellular hypertrophy and cell cycle arrest. Scale bar: 20um. **B**) Hyperactivation of YAP1 in cultured primary FTECs induced upregulated expression of factors associated with cell cycle arrest (P21 and P16). Please note that the presence of HPV16 E6/E7 eradicated YAP^{S127A}-induced upregulation of P21 and P16. **: P < 0.01 when compared to the control group (CTRL); ***: P < 0.001 when compared to the control group (CTRL); ****: P < 0.0001 when compared to the control group (CTRL).

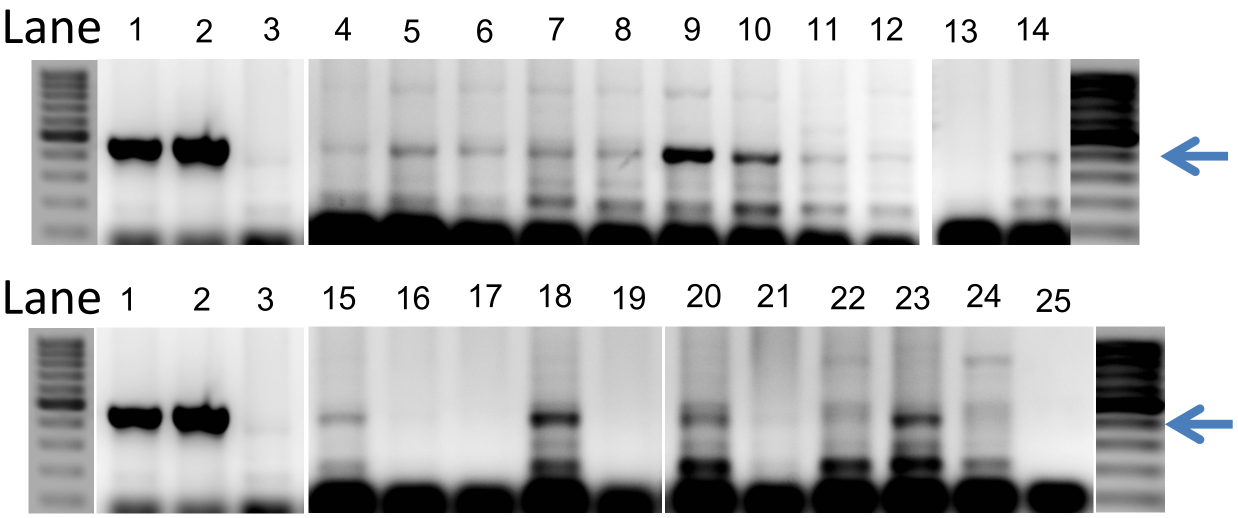

Appendix Figure S2. Representative images showing PCR products of HPV MY09/MY11 primer set using DNA extract from 24 cell lines.

Lanes: 1, ME180 cell; 2, HELA cell; 3, HT3 cell; 4, HOSET80 cell; 5, HOSE969 cell; 6, TOV21G cell; 7, SW626 cell; 8, SKOV3 cell; 9, IGROV1 cell; 10, OVCAR3 cell; 11, OVCAR5 cell; 12, OVCAR8 cell; 13, COV362 cell; 14, CAOV3 cell; 15, FT190 cell; 16, FT194 cell; 17, FT237 cell; 18, FT240 cell; 19, FT246 cell; 20, SK-UT-1 cell; 21, KLE cell; 22, AN3-CA cell; 23, MES-SA cell; 24, SK-LSM-1 cell; 25, H2O. H2O was used to replace the DNA template in reaction #25 (blank control). DNA extracted from HPV –positive ME180 cells (lane #1) and HELA cells (lane #2) were used as positive control. DNA extracted from HPV-negative HT3 cervical cancer cells. Experiment was repeated at least three times with three different DNA samples. Blue arrows on the right indicate the molecular weight of PCR products.

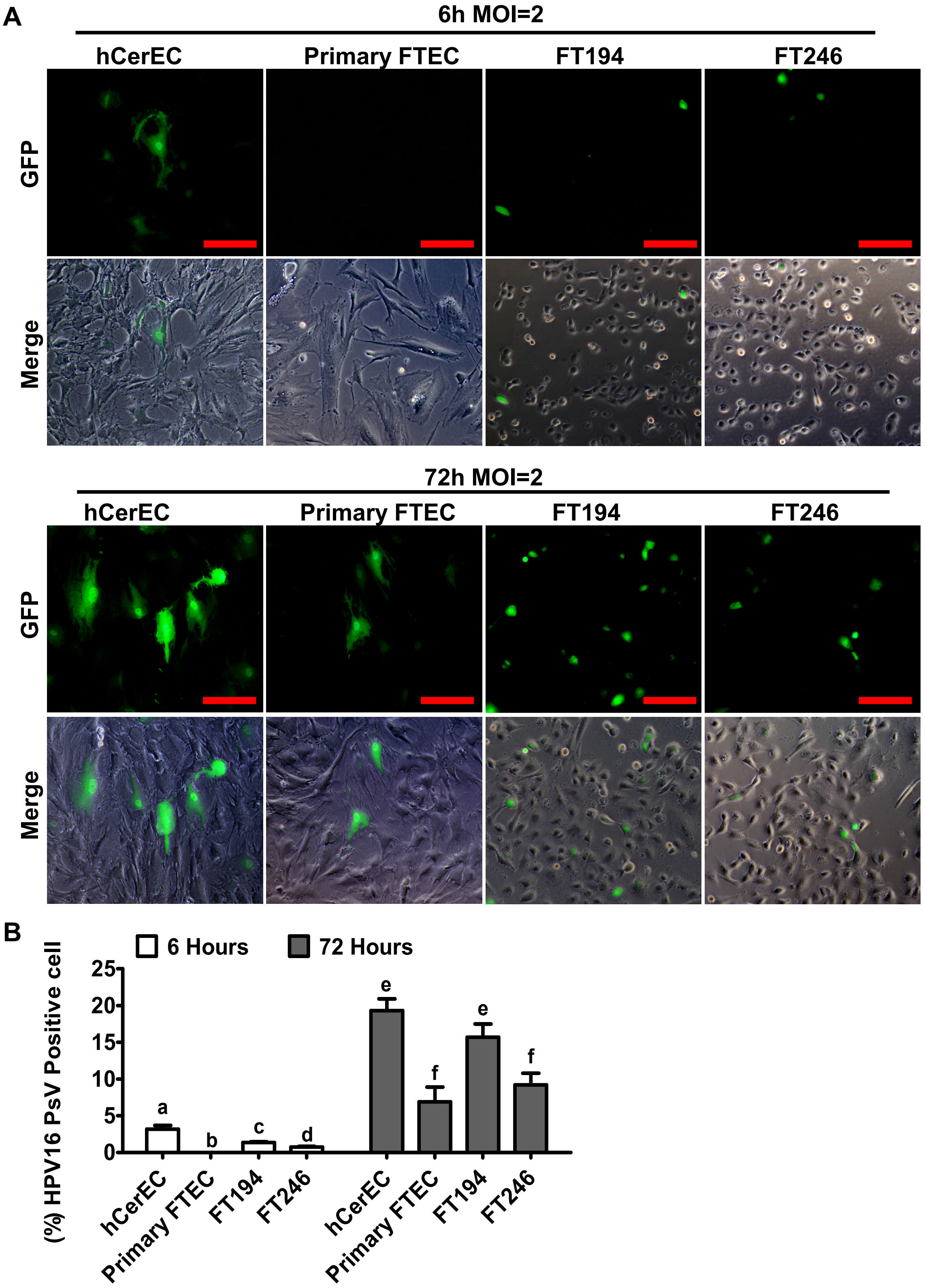

Appendix Figure S3. Effect of time and dosage on the infection efficiency of HPV16 PsV in fallopian tube epithelial cells.

A) Representative images showing HPV16 PsV GFP sinal in hCerEC (human cervical epithelial cells, positive control), primary FTECs, and two immortalized fallopian tube epithelial cells (FT194 and FT246 cells) after incubated with HPV16 PsV (MOI = 2, in 1 mL growth medium) for 6h (upper panel) or 72h (lower panel). Scale bar = 50 μm. **B**) Quantitative data showing percentage of HPV16 PsV positive cells in 72h groups. Each bar represents the mean ± SEM (n=4). Bars with different letters are significantly different from each other.

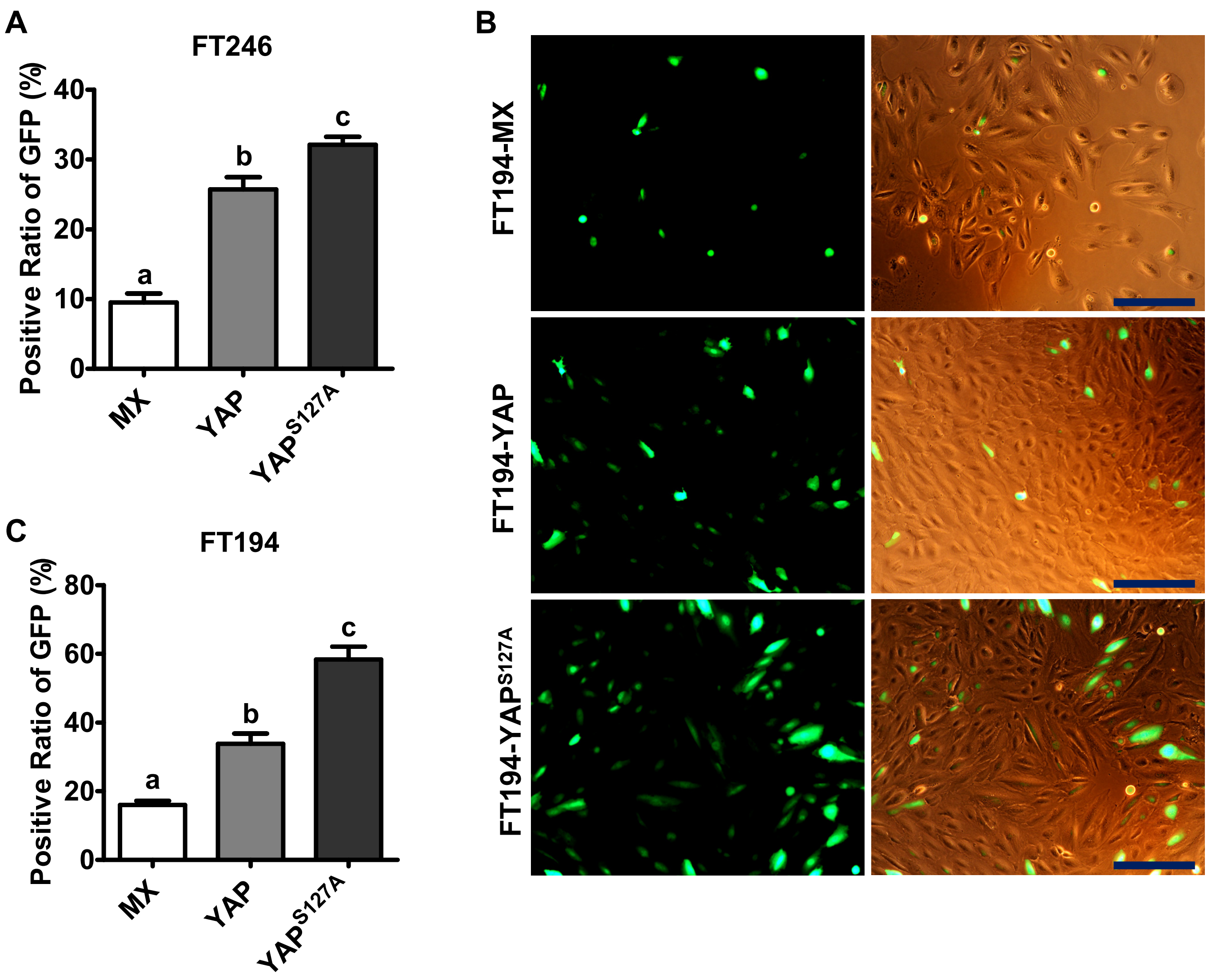

Appendix Figure S4. YAP increased susceptibility to HPV in FTECs.

A) Quantitative data showing the ratio of GFP positive cells in FT246-MX, FT246-YAP and FT246-YAP^{S127A} cells (corresponding to Figure 4A). Each bar represents the mean ± SEM (n=4). Bars with different letters are significantly (P < 0.01) different from each other. **B**) Representative images showing GFP expression in FT194-MX, FT194-YAP and FT194-YAP^{S127A} cells. **C**) Quantitative data showing the ratio of GFP positive cells in FT194-MX, FT194-YAP and FT194-YAP^{S127A} cells. Each bar represents the mean ± SEM (n=4). Bars with different letters are significantly (P < 0.01) different from each other.

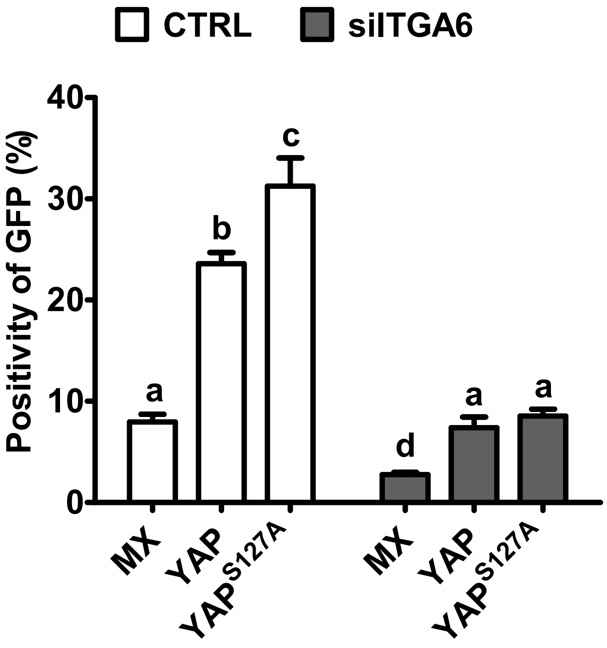

Appendix Figure S5. Knockdown of ITGA6 decreased YAP-induced susceptibility to HPV in FTECs.

Quantitative data showing the ratio of GFP (HPV16 PsV-GFP) positive cells in FT246-MX, FT246-YAP and FT246-YAP^{S127A} cells with or without ITGA6 knockdown (corresponding to Figure 3G). Each bar represents the mean ± SEM (n=4). Bars with different letters are significantly (P < 0.05) different from each other.

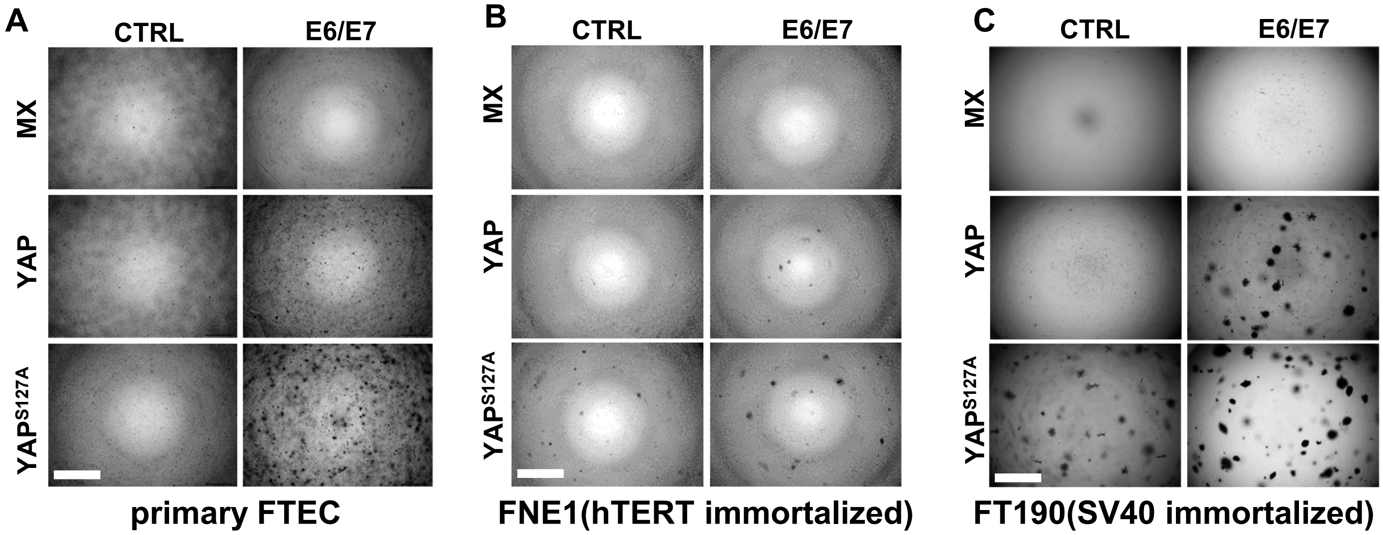

Appendix Figure S6. HPV E6/E7 contribute to YAP1-induced transformation of primary and immortalized fallopian tube epithelial cells.

A) Representative images from four independent experiments showing colony formation of primary FTEC-derived cells (FTEC-MX (control), FTEC-YAP, FTEC-YAP^{S127A}, FTEC-E6/E7, FTEC-E6/E7-YAP and FTEC-E6/E7-YAP^{S127A} cells) in the soft agar assay at their 6th passage. **B**) Representative images from four independent experiments showing colony formation of primary FTEC-derived cells (FNE1-MX (control), FNE1-YAP, FNE1-YAP^{S127A}, FNE1-E6/E7, FNE1-E6/E7-YAP and FNE1-E6/E7-YAP^{S127A} cells) in the soft agar assay at their 6th passage. Representative images showing colony formation in the soft agar system at their 6rd passage. **C**) Representative images from four independent experiments showing colony formation of primary FTEC-derived cells (FT190-MX (control), FT190-YAP, FT190-YAP^{S127A}, FT190-E6/E7, FT190-E6/E7-YAP and FT190-E6/E7-YAP^{S127A} cells) in the soft agar assay at their 6th passage. Scale bar = 1000μm.

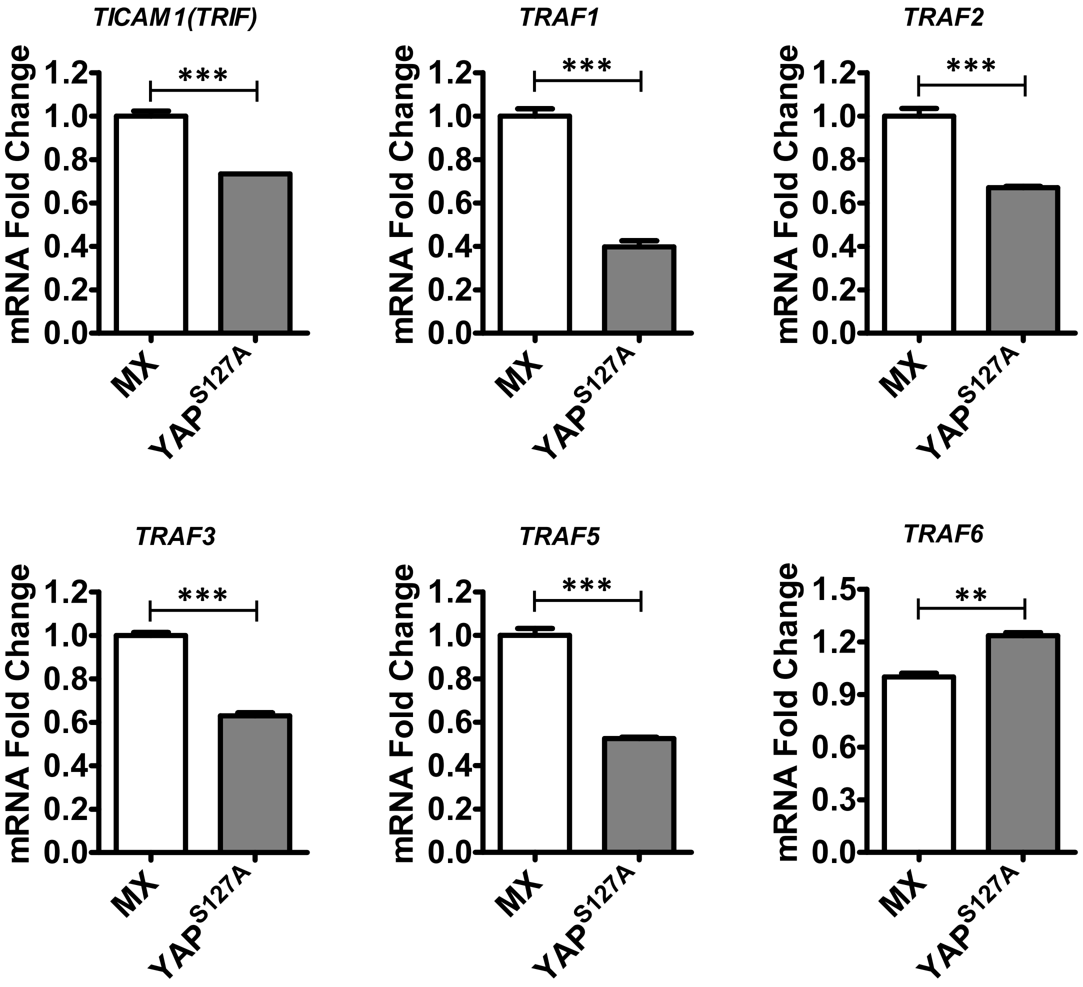

Appendix Figure S7. Constitutive activation of YAP inhibited viral recognition of HPV infection in FTECs.

Quantitative data showing mRNA expressions of TLRs adaptor receptors and TNF receptor associated factors in FNE1-MX and FNE1-YAP^{S127A} cells. Each bar represents the mean ± SEM (n = 3). ** : *P* < 0.01, compared with MX control; *** : *P* < 0.001, compared with MX control (MX).

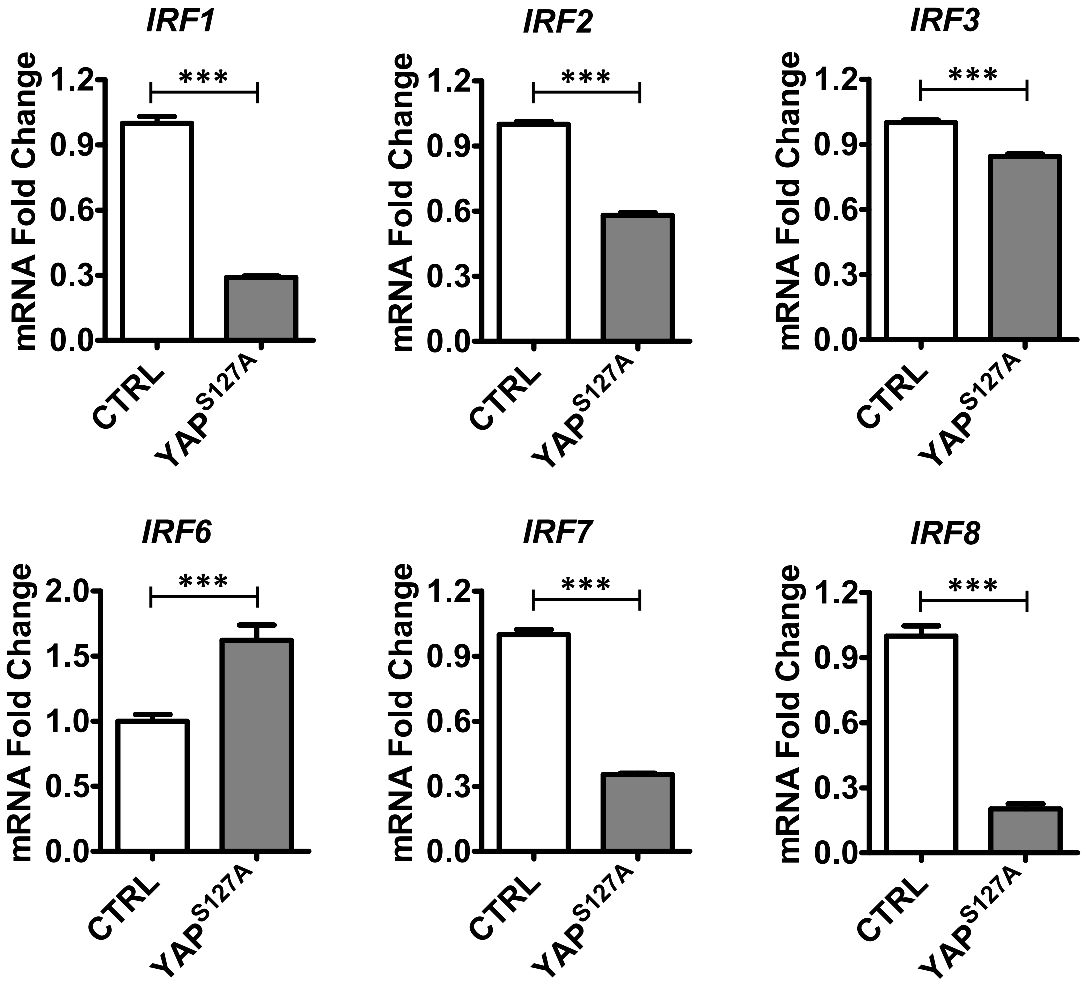

Appendix Figure S8. Constitutive activation of YAP inhibited the expression of interferon regulatory factors.

Quantitative data showing the mRNA expressions of Interferon regulatory factors in FNE1-MX and FNE1-YAP^{S127A} cells. Each bar represents the mean ± SEM (n=4). *** : *P* < 0.001, compared with MX control (CTRL).

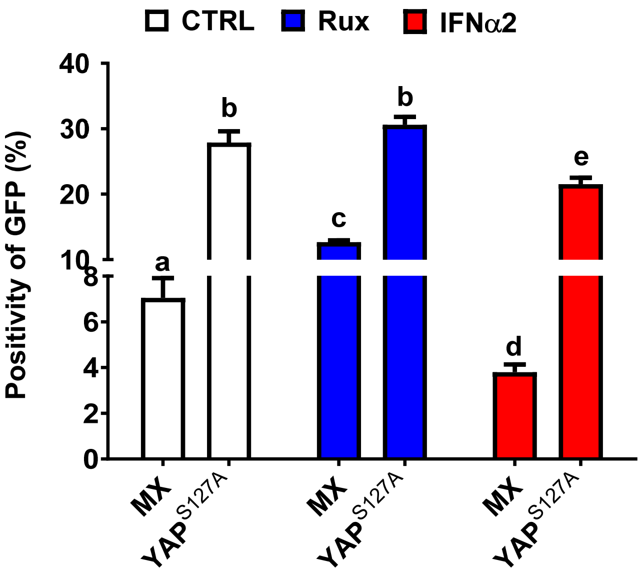

Appendix Figure S9. Constitutive activation of YAP suppressed the IFNαRs / JAK / STATs signaling pathway.

Quantitative data showing the ratio of GFP positive cells in FT246-MX and FT246-YAP^{S127A} cells treated with or without Ruxolitinib (Rux) and IFNα2 in corresponding to Figure 7D. IFNα2b greatly reduced the ration of GFP positive cells in FT246-Mx control cells, but not in FT246-YAP^{S127A} cells. Each bar represents the mean ± SEM (n=4). Bars with different letters are significantly (P < 0.05) different from each other.

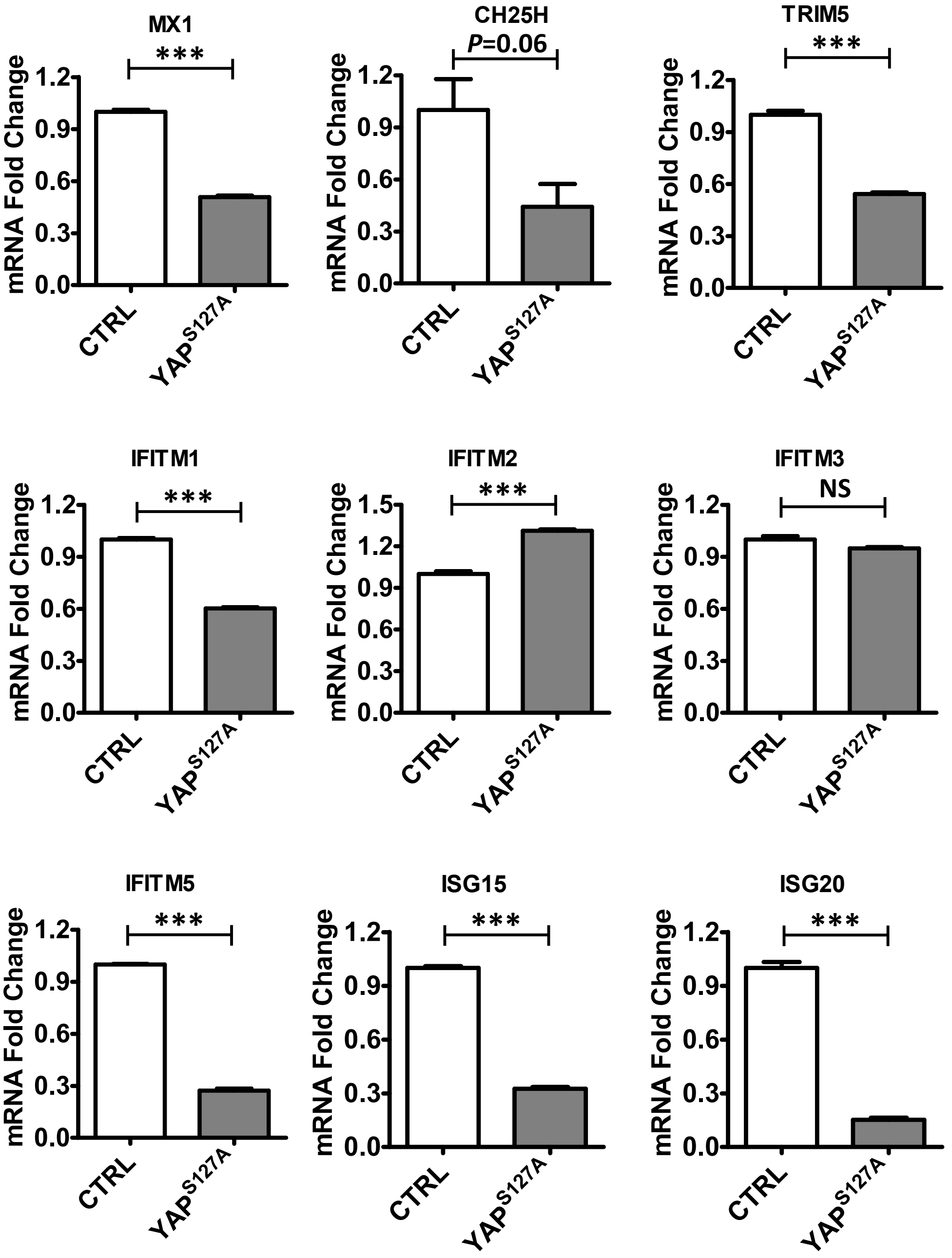

Appendix Figure S10. Constitutive activation of YAP inhibited the productions of interferon-stimulated genes (ISGs) in FTECs.

Quantitative data showing the mRNA expressions of some important ISGs in FNE1-MX and FNE1-YAP^{S127A} cells. Each bar represents the mean ± SEM (n=4). NS : No significant difference compared to MX control (CTRL); *** : $P < 0.001$, compared with MX control (CTRL).

Appendix table S1. Sequences of primers used in RT-PCR analyses

Genes	Forward primer sequences (5' to 3')	Reverse primer sequences (5' to 3')
TLR1	GGTCTTGCTGGTCTTAGGAGAGAC	CTGAAGTCCAGCTGACCCTGTAGCTTCACG
TLR2	GGCCAGCAAATTACCTGTGTG	CTGAGCCTCGTCCATGGGCCACTCC
TLR3	CGGGCCAGCTTTCAGGAACCTG	GGCATGAATTATATATGCTGC
TLR4	TGCAATGGATCAAGGACCAGAGGC	GTGCTGGGACACCACAACAATCACC
TLR5	CCTCATGACCATCCTCACAGTCAC	GGCTTCAAGGCACCAGCCATCTC
TLR6	CCAAGTGAACATATCAGTTAATACTTTAGGGTGC	CTCAGAAAACACGGTGTACAAAGCTG
TLR7	CTCCCTGGATCTGTACACCTGTGAG	CTCCCACAGAGCCTTTTCCGGAGCT
TLR7II	TTTACCTGGATGGAAACCAGCTA	TCAAGGCTGAGAAGCTGTAAGCTA
TLR8	GTCCTGGGGATCAAAGAGGGAAGAG	CTCTTACAGATCCGCTGCCGTAGCC
TLR9	GCGAGATGAGGATGCCCTGCCCTACG	TTCGGCCGTGGGTCCCTGGCAGAAG
TLR10	CAGAGGTCATGATGGTTGGATGG	GACCTAGCATCCTGAGATACCAGGGCAG
MyD88	CGGCAACTGGAGACACAAG	TCTGGAAGTCACATTCCTTGC
TRIF	GCAGCCCCGGATCCCT	TGTCCCCTACCCATTCACTGTT
TICAM1	GCACCAACTACCCAGTGGAG	TGGCGTCTGGTCTTTGACAG
IRF1	GCCAGTCGACGAGGATGAGGAAGGGAA	CCAGCGGCCGCCTGCTACGGTGCACAGG
IRF2	CCAGTCGACTACCTCAGCAACATGGGG	CCAGCGGCCGCGGCTTAACAGCTTGAC
IRF3	CGGAAGCTTCTGAAGCGGCTGTTGGTG	GTGCTCGAGACCATGAGGAGCGAGGGC
IRF4	CCAGTCGACGCAAGCTCTTTGACACAC	CCAGCGGCCGCCTTTTCATTCTTGAATAG
IRF5	GCCTTGTTATTGCATGCCAGC	AGACCAAGCTTTTCAGCCTGG
IRF7	TGCAAGGTGTACTGGGAG	TCAAGCTTCTGCTCCAGCTCCATAAG
IRF8	GCCGAATTCTCCGAGAGCTGCAGCA	CGGCTCGAGGCTTAGACGGTGATC
IRF9	TTCTGTCCCTGGTGTAGAGCCT	TTTCAGGACACGATTATCACGG
NFkB1	CACTGCTCAGGTCCACTGTC	CTGTCACTATCCCGGAGTTCA
NFKB2	GGGGCATCAAACCTGAAGATTTCT	TCCGGAACACAATGGCATACTGT
IFNA	GTACTGCAGAATCTCTCCTTTCTCCTG	GTGTCTAGATCTGACAACCTCCCAGGCACA
IFNA2	GTACTGCAGAATCTCTCTTTTCTCCTG	GTGTCTAGATCTGACAACCTCCCAGGCACA
IFNB	TTGTGCTTCTCCACTACAGC	CTGTAAGTCTGTTAATGAAG-
IFNE	TCTTGATTCACTTGTCTTTGCTG	CCTTCACCATGATTATCAAGCAC
FNAR1	AGTGTTATGTGGGCTTTGGATGGTTTAAGC	TCTGGCTTTCACACAATATACAGTCAGTGG
FNAR2	AGTGTTATGTGGGCTTTGGATGGTTTAAGC	CACTTTCTTCTTTCTGTTGA
IFNAR1	CAGCACCTGATGGCCTATCAC	TGGAGCATGAAGAACTGGATG
IFNAR2	CATCCACCTGAATGCCTACTTCT	TCTGCAAGGAGTCACCATTCTCT
STAT1	GGCACCAGAACGAATGAGGG	CCATCGTGCACATGGTGGAG
STAT2	GCAGCACAATTTGGGAA	ACAGGTGTTTCGAGAACTGGC
TRAF1	GGAGGCATCCTTTGATGGTA	AGGGACAGGTGGGTCTTCTT
TRAF2	TTCCCCTTAACTTGTGACGGC	CAATCTTGTCTTGGTCCAGCC
TRAF3	CTCACAAGTGCAGCGTCCAG	GCTCCACTCCTTCAGCAGGTT
TRAF4	CTGGCTAAACCACAGCACGTC	TCGCTTTCGAATGTCCTGG
TRAF5	CTGTGCTGTAACGGATAAACGG	TAGCTGCTGGATTTTACTTTCTTTC
TRAF6	TACGAGAAGCAGTGCAAACGC	ATTTTTGGAAGGGACGCTGGC
CH25H	ATCACCACATACGTGGGCTTT	GTCAGGGTGGATCTTGTAGCG
TRIM5	AGGAGTTAAATGTAGTGCT	ACCATGGATTTCTCATCTAT
IFI44L	GCTGCGGGCTGCAGAT	CTCTCTCAATTGCACCAGTTTCC
RSAD2	CTTTGTGCTGCCCCTTGAG	TCCATACCAGCTTCCTTAAGCAA
IFI27	GGCAGCCTTGTGGCTACTCT	CCCAGGATGAACTTGGTCAATC
USP18	CTCAGTCCCGACGTGGAACT	ATCTCTCAAGCGCCATGCA
HERC6	CACTACCACTCCCTGGCATT	TGTTACTTCCCCAGCCAAAV
MXA	CAAATACCTGACTGTGAAAATGTCAAA	CAAACTTTATCTCTTCAGACCAAAAAGA
MX1	GTGCATTGCAGAAGGTCAGA	TCAGGAGCCAGCTTAGGTGT
ISG15	TCCTGCTGGTGGTGGACAA	TTGTTATTCCTCACCAGGATGCT
ISG20	ATCTCTGAGGGTCCCCAAGGA	TTCAGTCTGACACAGCCAGGCG
OAS1	TGATGCCCTGGGTCAGTTG	TCGGTGCACTCCTCGATGA
OAS1	TGAGGTCCAGGCTCCACGCT	GCAGGTCGGTGCACTCCTCG
OAS2	ACAGCTGAAAGCCTTTTGGA	AAGTTTCGCTGCAGGACTGT
LGALS3BP	GGCTGGCTGAAGAGCAACTG	GTGGGTGCTCCTGGTTTCAT
IFIT1	TCTCAGAGGAGCCTGGCTAA	CCAGACTATCCTTGACCTGATGA
IFITM1	GGATTTCGGCTTGTCCCGAG	CCATGTGGAAGGGAGGGCTC
IFITM2	ATTGTGCAAACCTTCTCTCCTG	ACCCCCAGCATAGCCACTTCCT
IFITM3	ACTGTCCAAACCTTCTTCTCTC	AGCACAGCCACCTCGTGCTC
IFITM5	TTGATCTGGTCGGTGTTCAG	GTCAGTCATAGTCCGCGTCA
