# Supplementary figures and images for: HPV-YAP1 oncogenic alliance drives malignant transformation of fallopian tube epithelial cells
Source: EMBO Rep. 2024 Sep 13;25(10):26. doi: 10.1038/s44319-024-00233-3 (PMC11467260; doi:10.1038/s44319-024-00233-3)

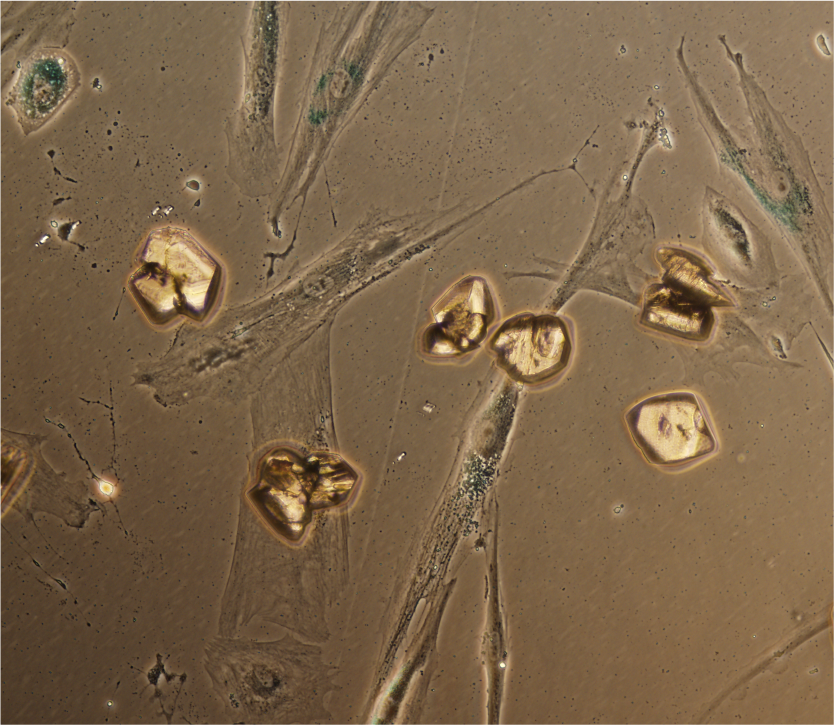

Supplement: Supplementary file 3 — Source data Fig. 1 [file 44319_2024_233_MOESM3_ESM.zip › Figure 1 Source data - zip/Figure 1A-1.tif]

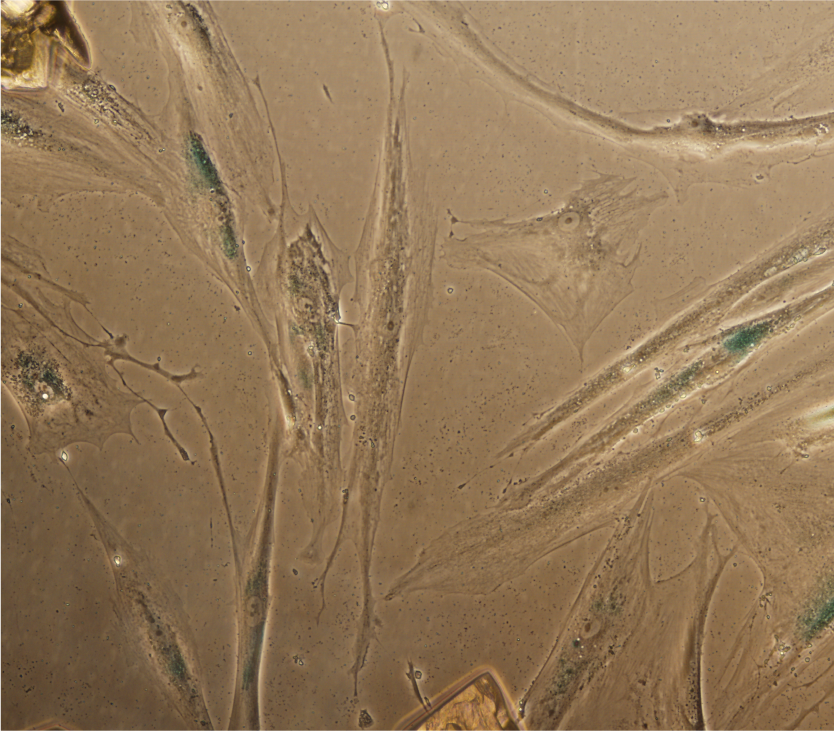

Supplement: Supplementary file 3 — Source data Fig. 1 [file 44319_2024_233_MOESM3_ESM.zip › Figure 1 Source data - zip/Figure 1A-2.tif]

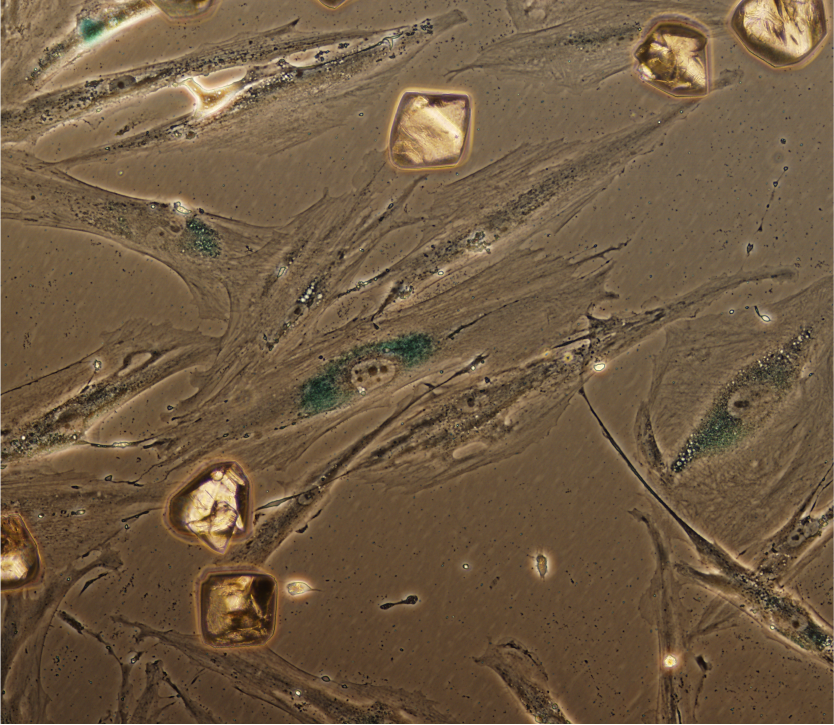

Supplement: Supplementary file 3 — Source data Fig. 1 [file 44319_2024_233_MOESM3_ESM.zip › Figure 1 Source data - zip/Figure 1A-3.tif]

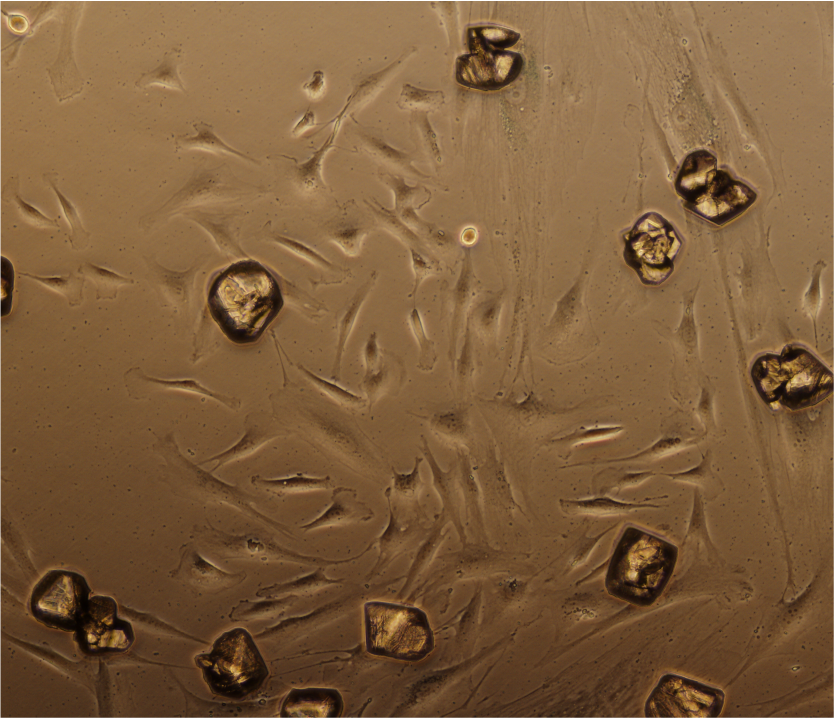

Supplement: Supplementary file 3 — Source data Fig. 1 [file 44319_2024_233_MOESM3_ESM.zip › Figure 1 Source data - zip/Figure 1A-4.tif]

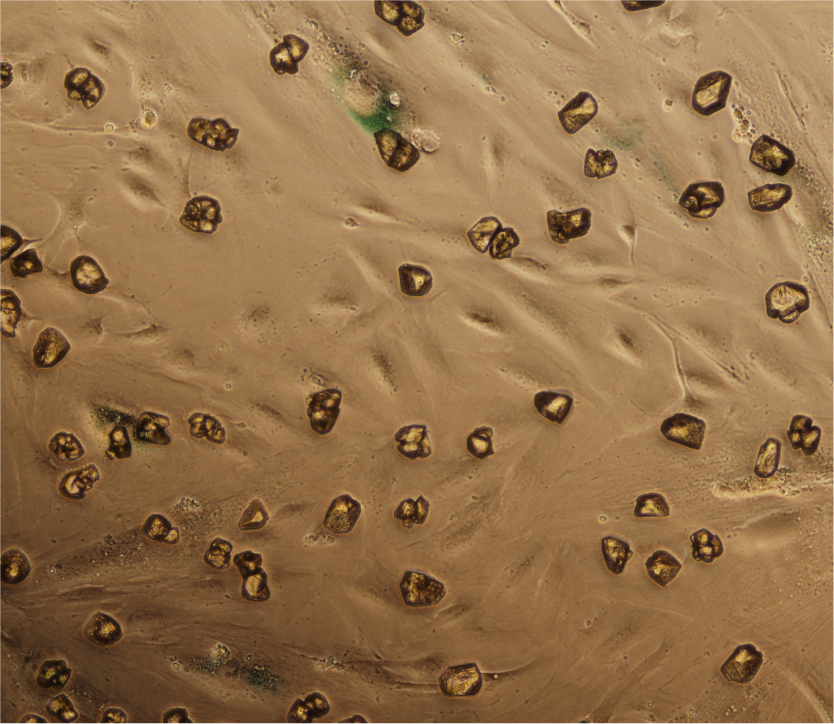

Supplement: Supplementary file 3 — Source data Fig. 1 [file 44319_2024_233_MOESM3_ESM.zip › Figure 1 Source data - zip/Figure 1A-5.tif]

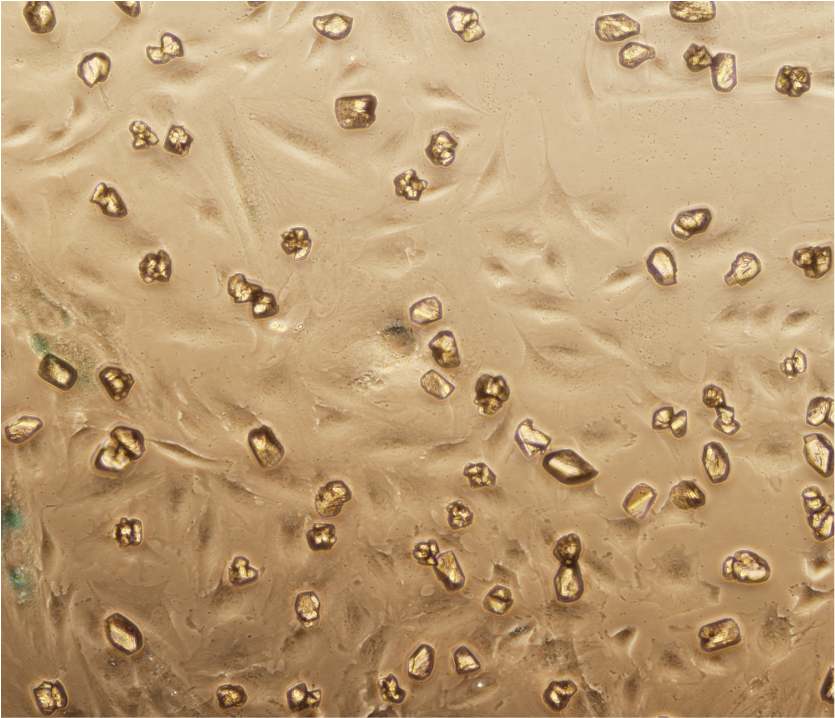

Supplement: Supplementary file 3 — Source data Fig. 1 [file 44319_2024_233_MOESM3_ESM.zip › Figure 1 Source data - zip/Figure 1A-6.tif]

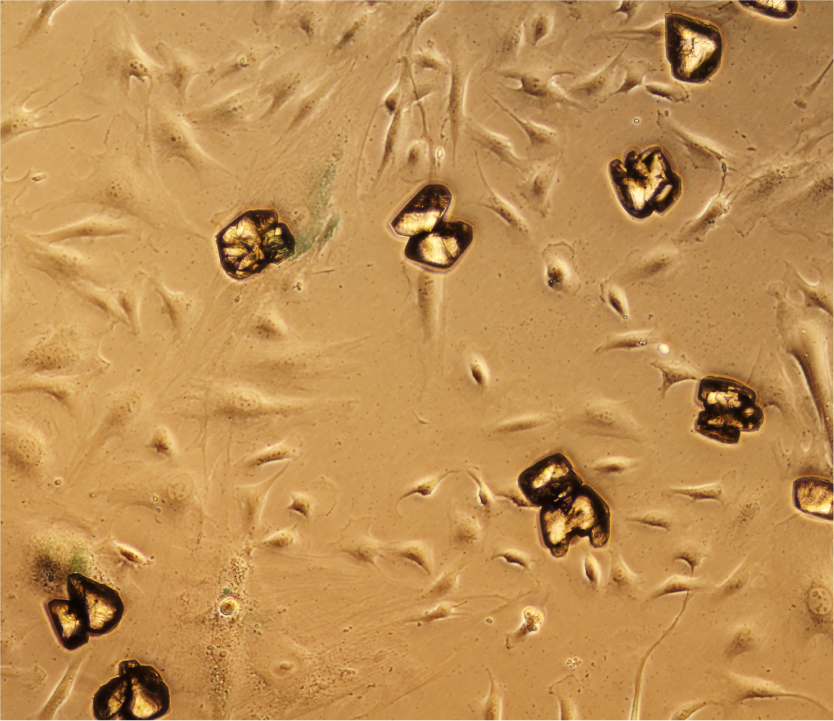

Supplement: Supplementary file 3 — Source data Fig. 1 [file 44319_2024_233_MOESM3_ESM.zip › Figure 1 Source data - zip/Figure 1C-1.tif]

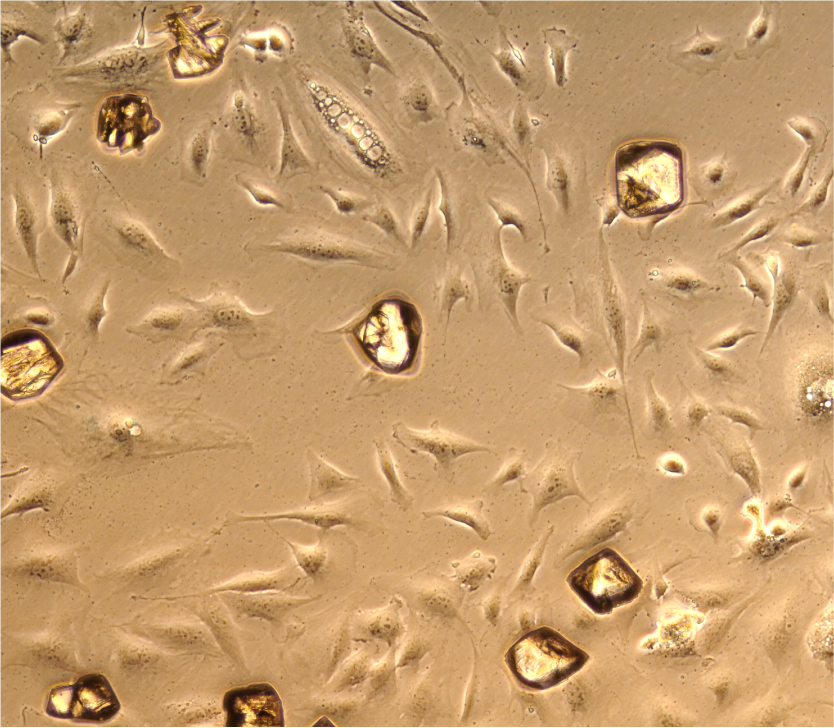

Supplement: Supplementary file 3 — Source data Fig. 1 [file 44319_2024_233_MOESM3_ESM.zip › Figure 1 Source data - zip/Figure 1C-2.tif]

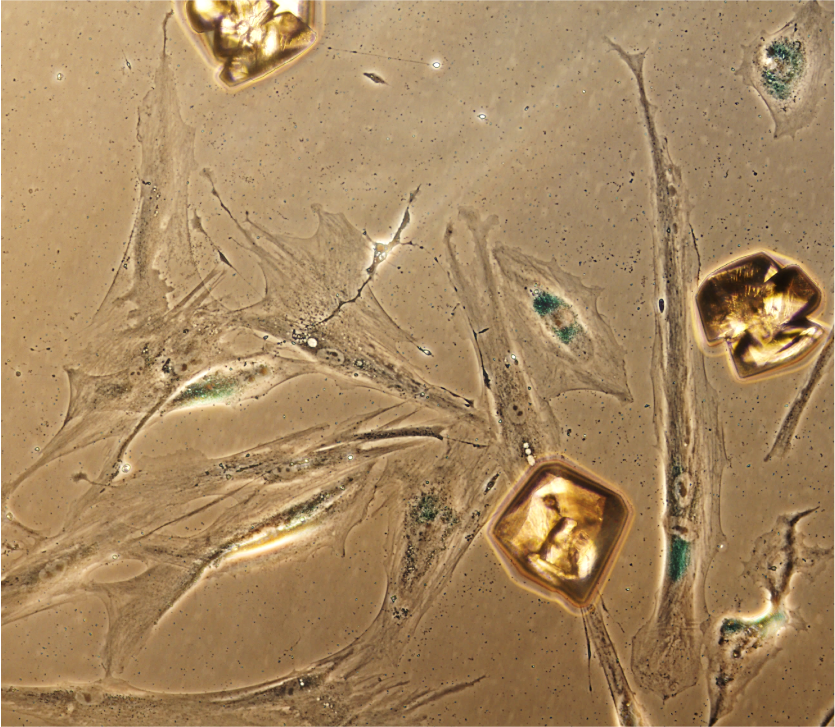

Supplement: Supplementary file 3 — Source data Fig. 1 [file 44319_2024_233_MOESM3_ESM.zip › Figure 1 Source data - zip/Figure 1E-1.tif]

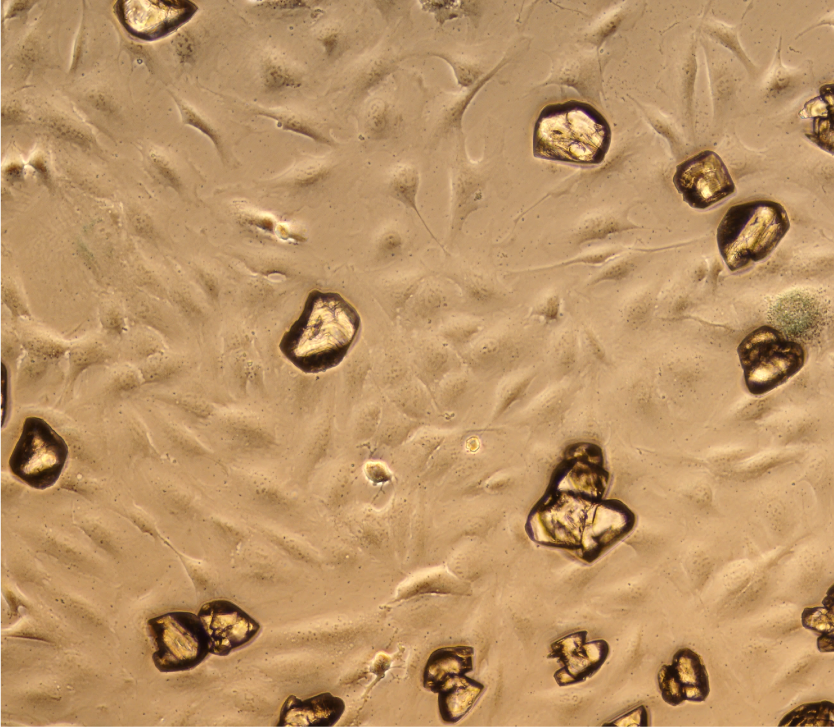

Supplement: Supplementary file 3 — Source data Fig. 1 [file 44319_2024_233_MOESM3_ESM.zip › Figure 1 Source data - zip/Figure 1E-2.tif]

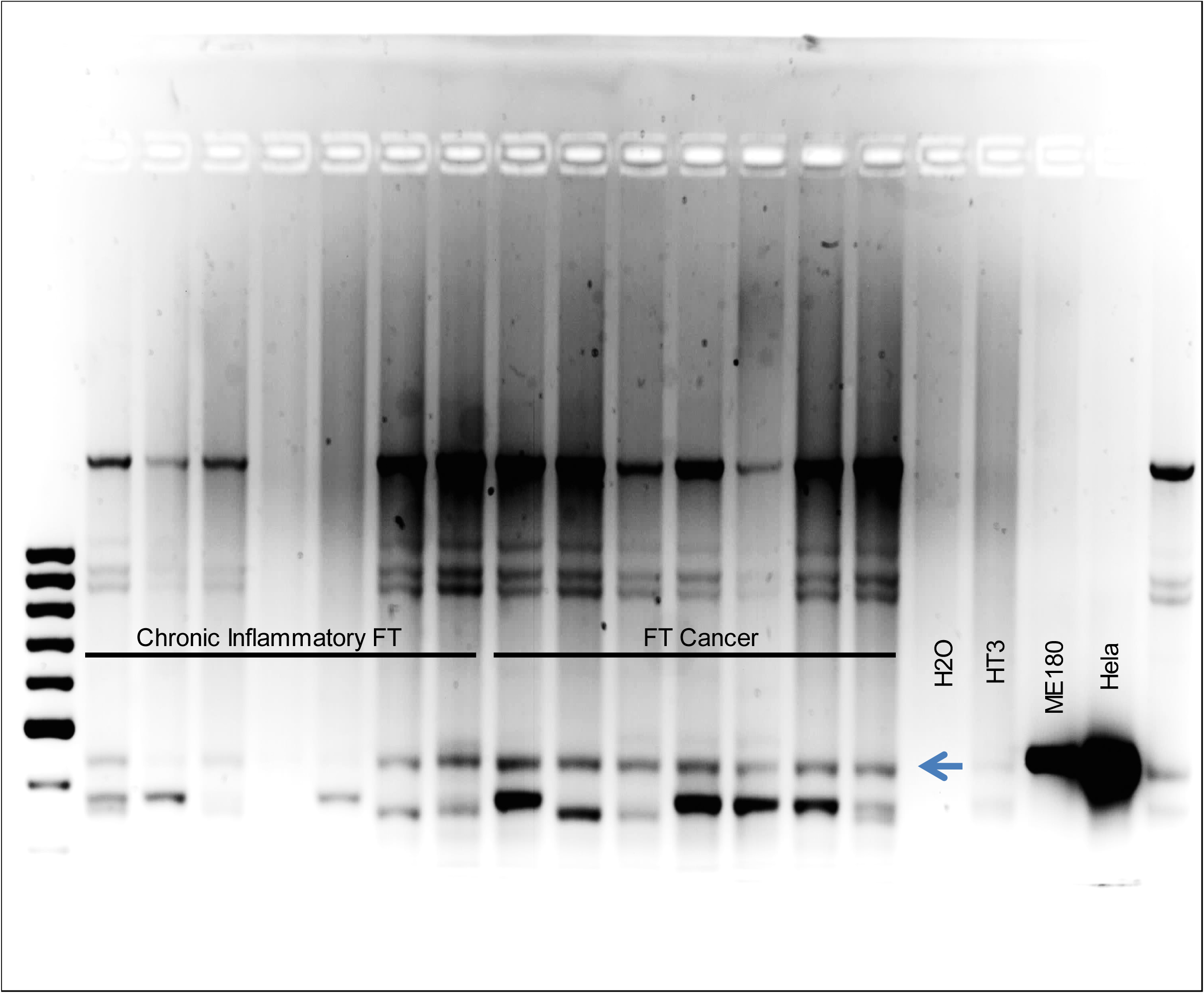

Supplement: Supplementary file 4 — Source data Fig. 2 [file 44319_2024_233_MOESM4_ESM.zip › Figure 2 Source data - zip/Figure 2A.tif]

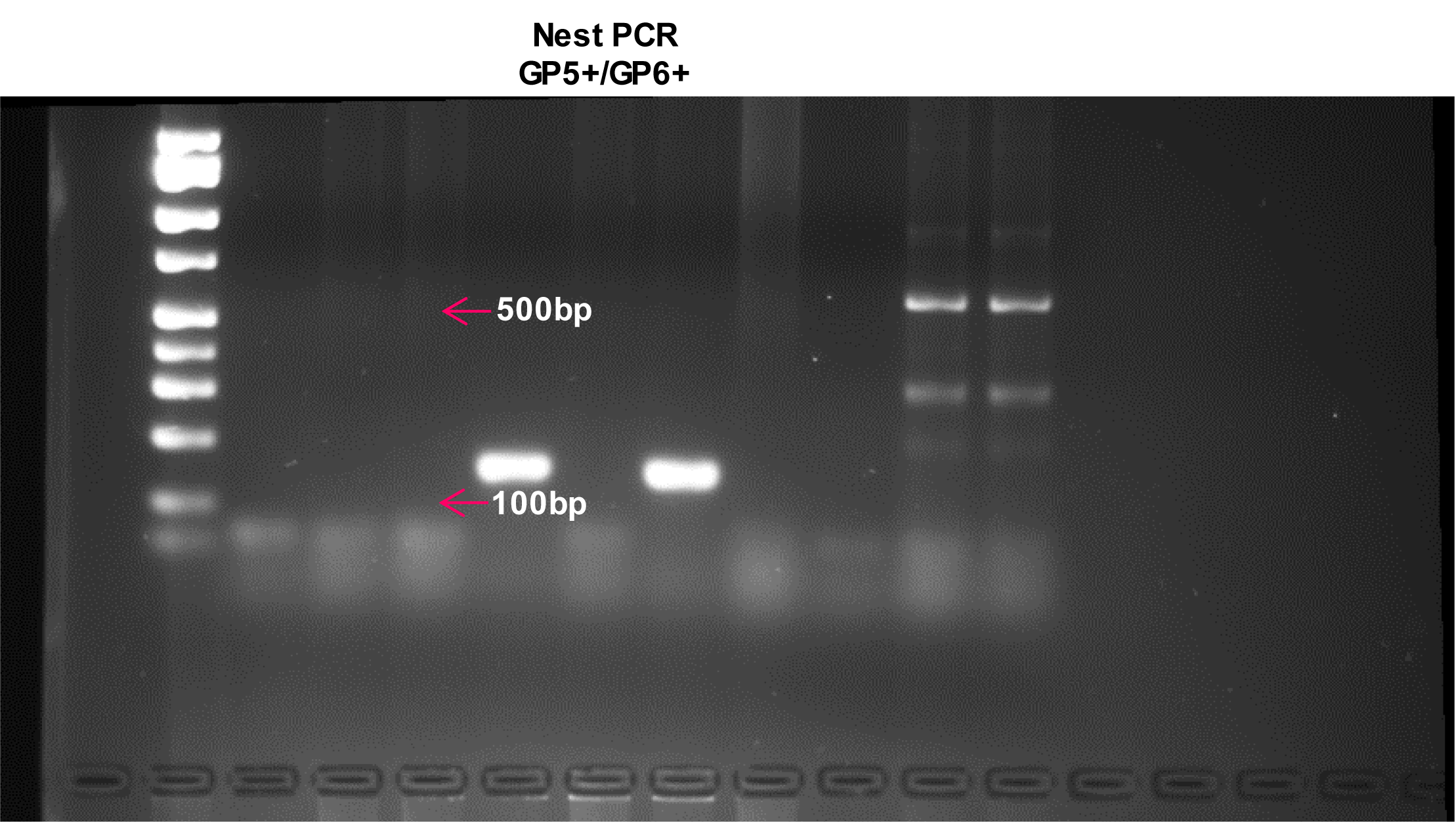

Supplement: Supplementary file 4 — Source data Fig. 2 [file 44319_2024_233_MOESM4_ESM.zip › Figure 2 Source data - zip/Figure 2D.tif]

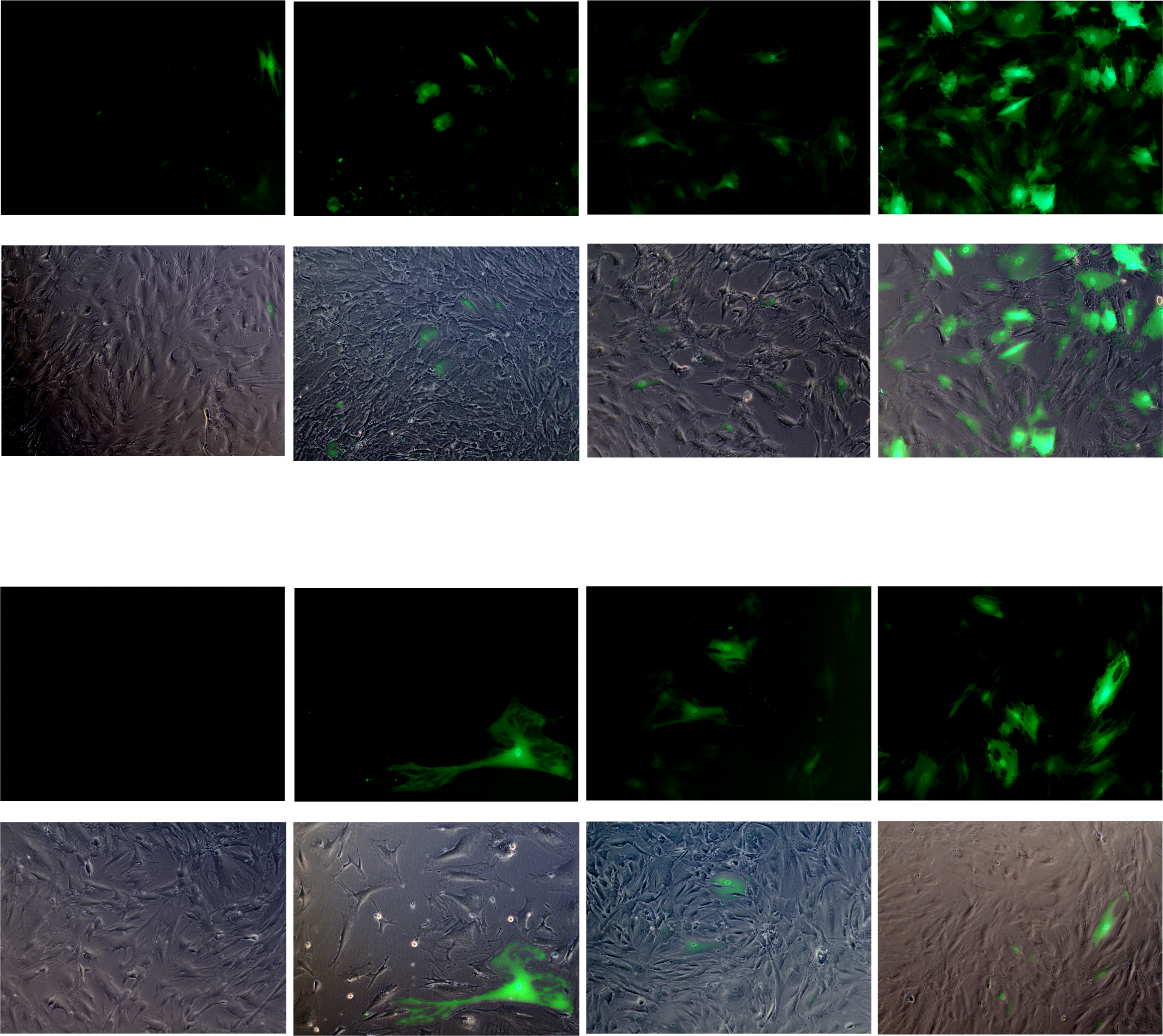

Supplement: Supplementary file 5 — Source data Fig. 3 [file 44319_2024_233_MOESM5_ESM.zip › Figure 3 Source data - zip/Figure 3A.tif]

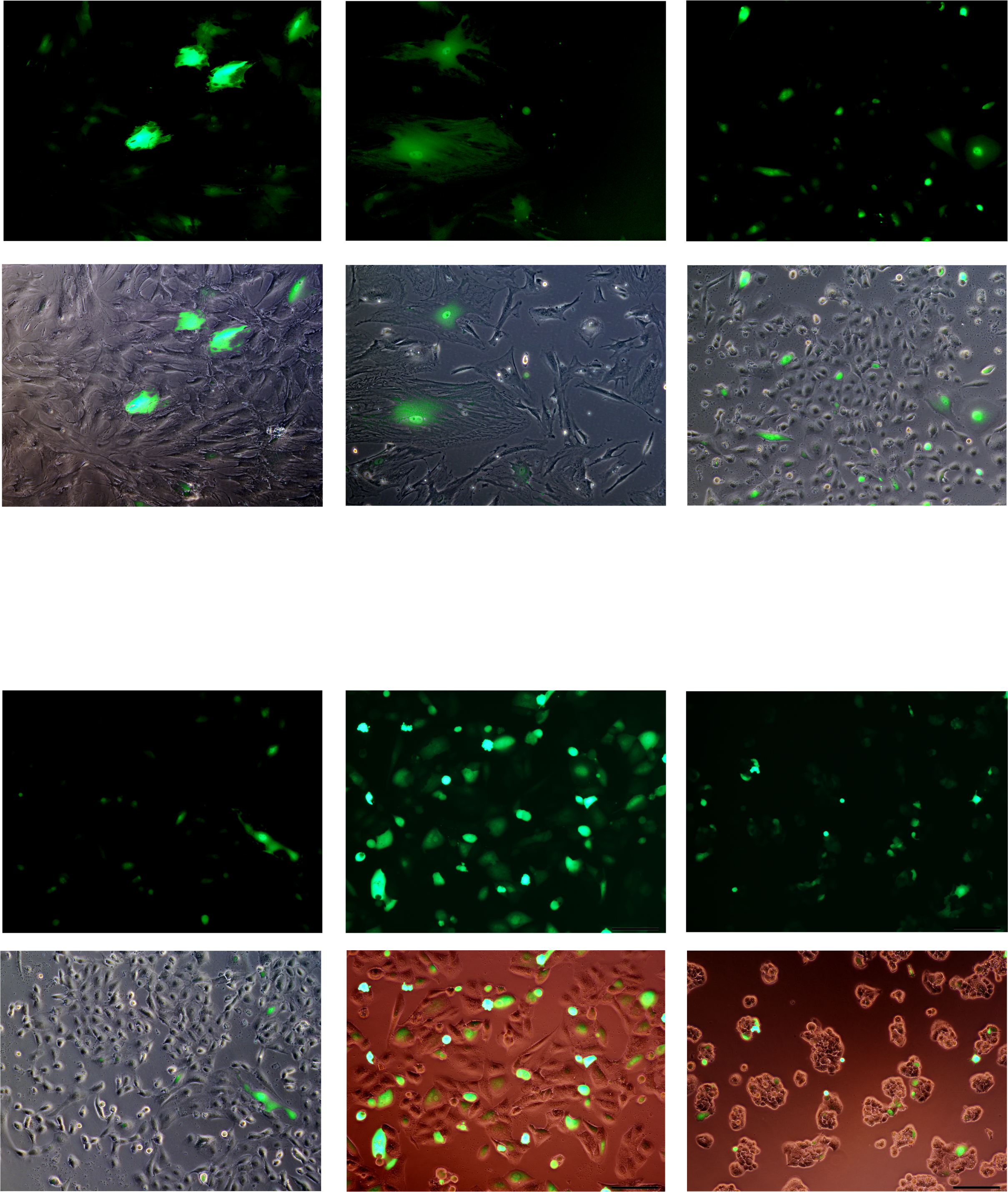

Supplement: Supplementary file 5 — Source data Fig. 3 [file 44319_2024_233_MOESM5_ESM.zip › Figure 3 Source data - zip/Figure 3C.tif]

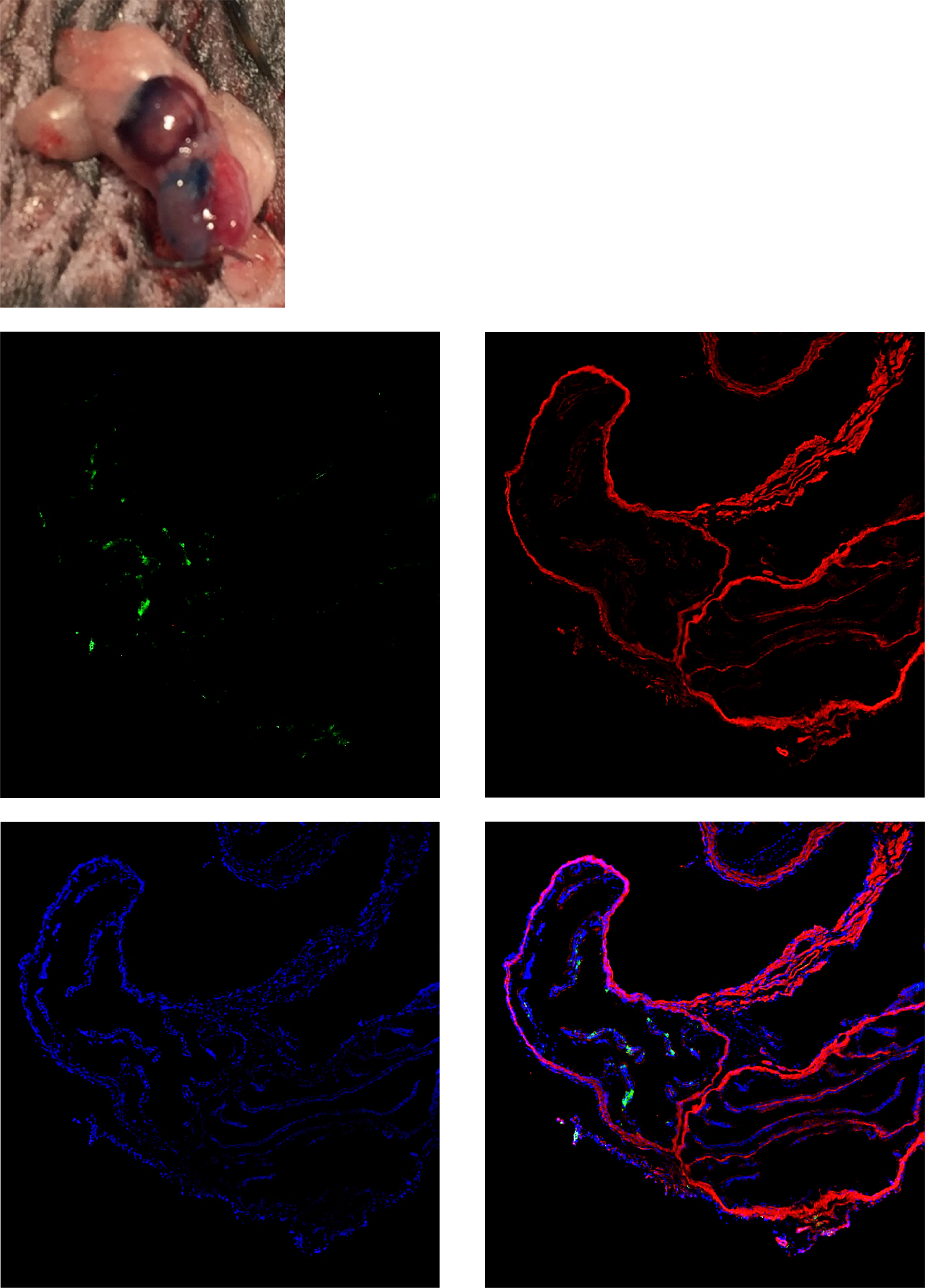

Supplement: Supplementary file 5 — Source data Fig. 3 [file 44319_2024_233_MOESM5_ESM.zip › Figure 3 Source data - zip/Figure 3F.tif]

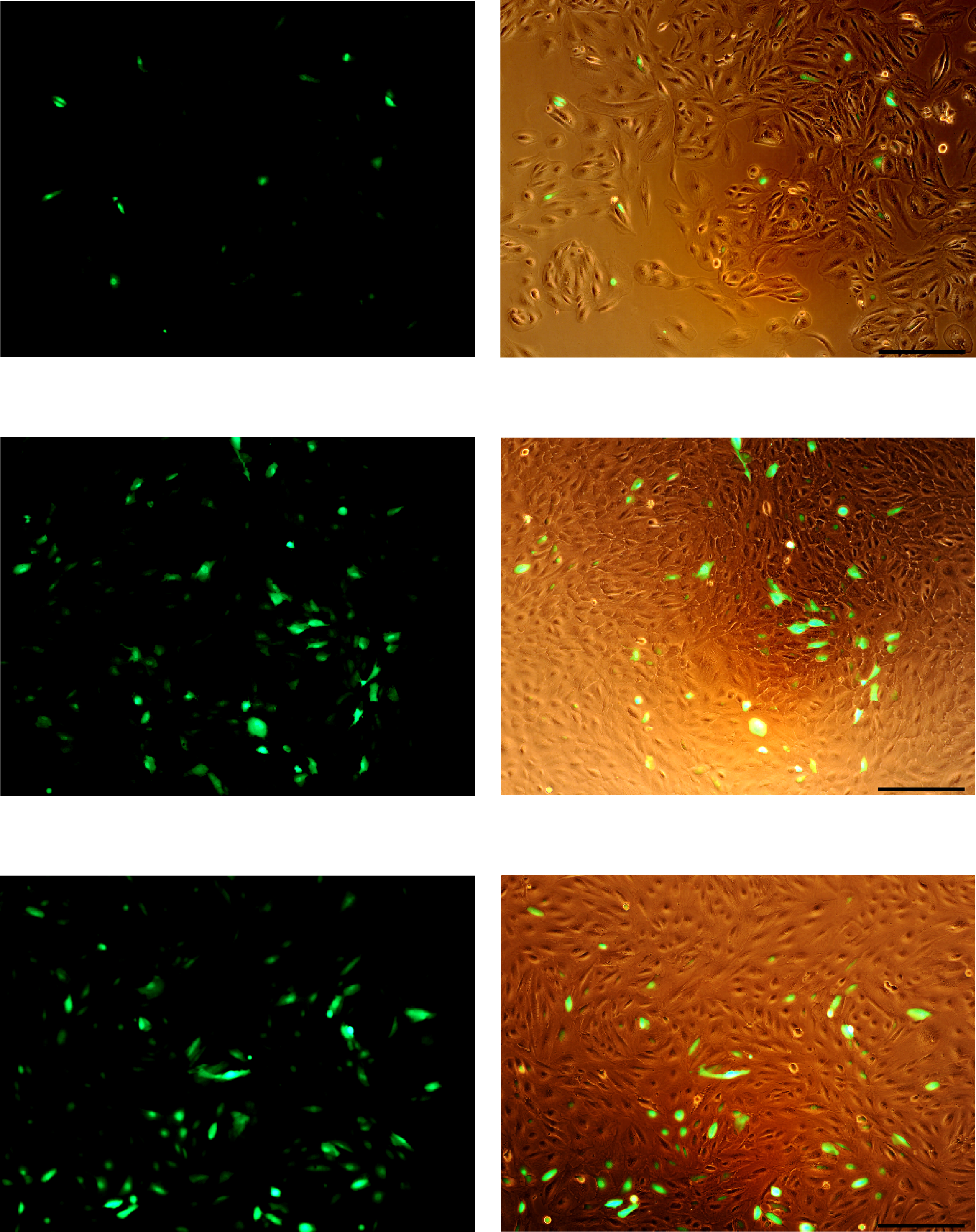

Supplement: Supplementary file 6 — Source data Fig. 4 [file 44319_2024_233_MOESM6_ESM.zip › Figure 4 Source data - zip/Figure 4A.tif]

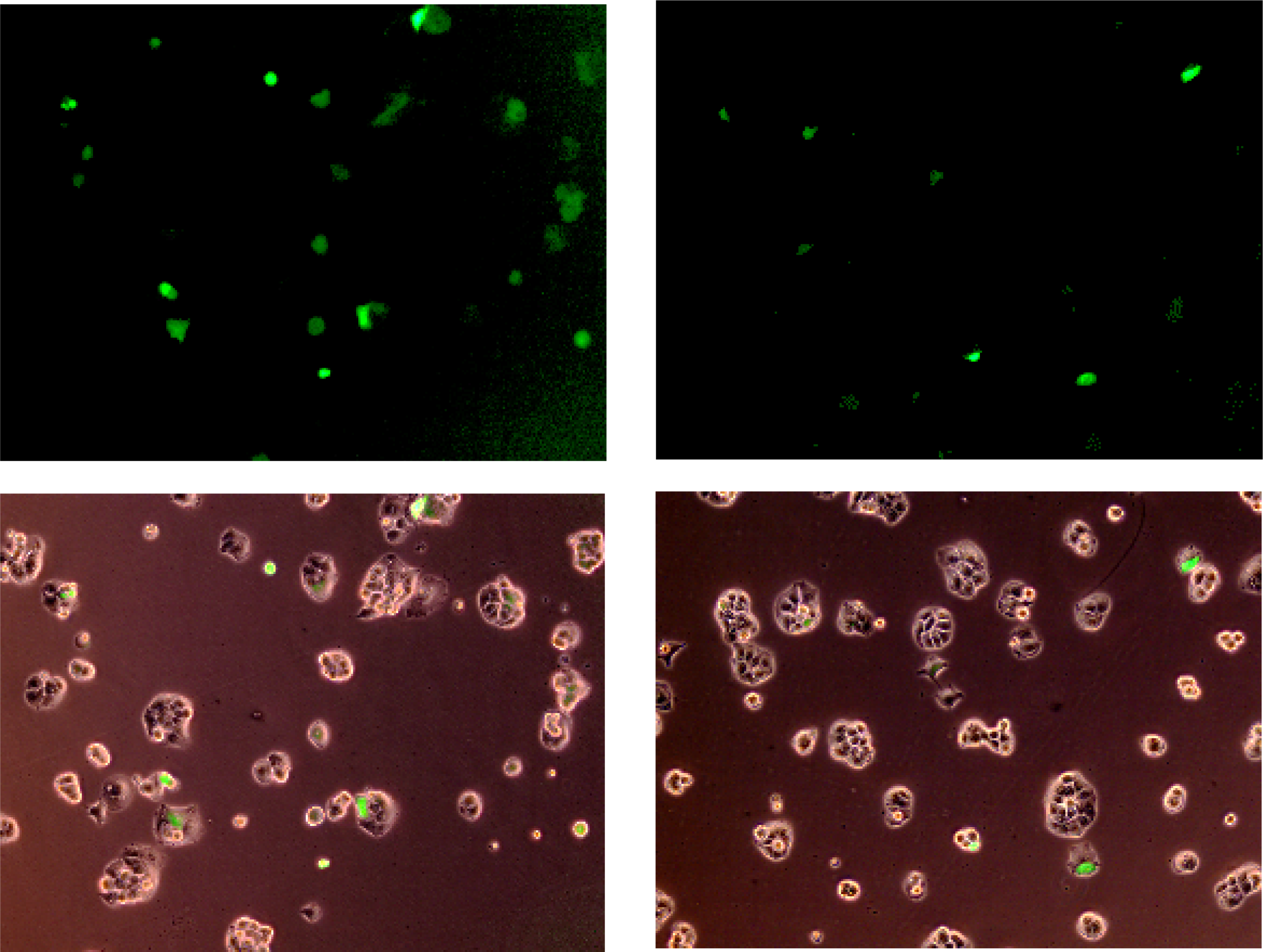

Supplement: Supplementary file 6 — Source data Fig. 4 [file 44319_2024_233_MOESM6_ESM.zip › Figure 4 Source data - zip/Figure 4E.tif]

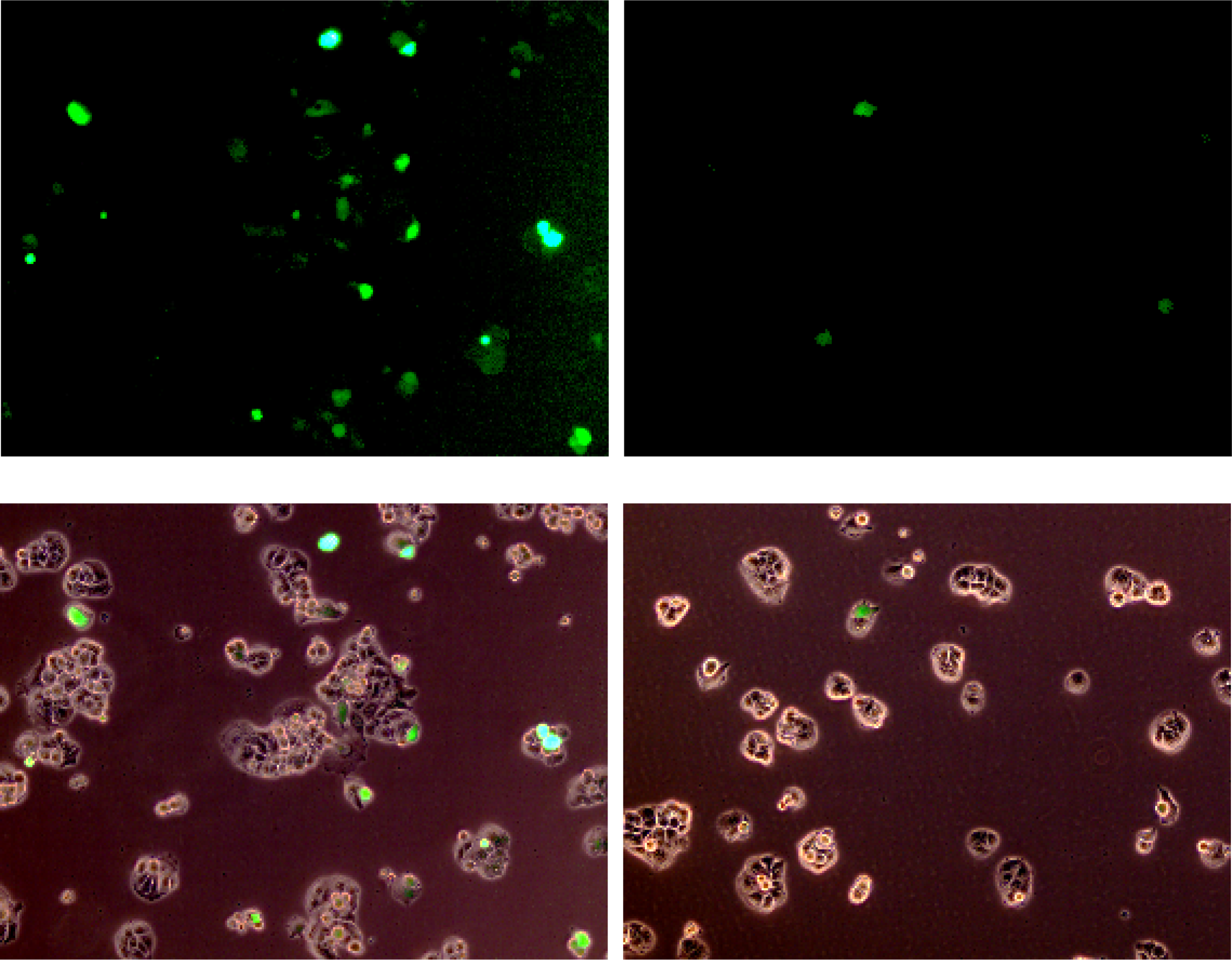

Supplement: Supplementary file 6 — Source data Fig. 4 [file 44319_2024_233_MOESM6_ESM.zip › Figure 4 Source data - zip/Figure 4G.tif]

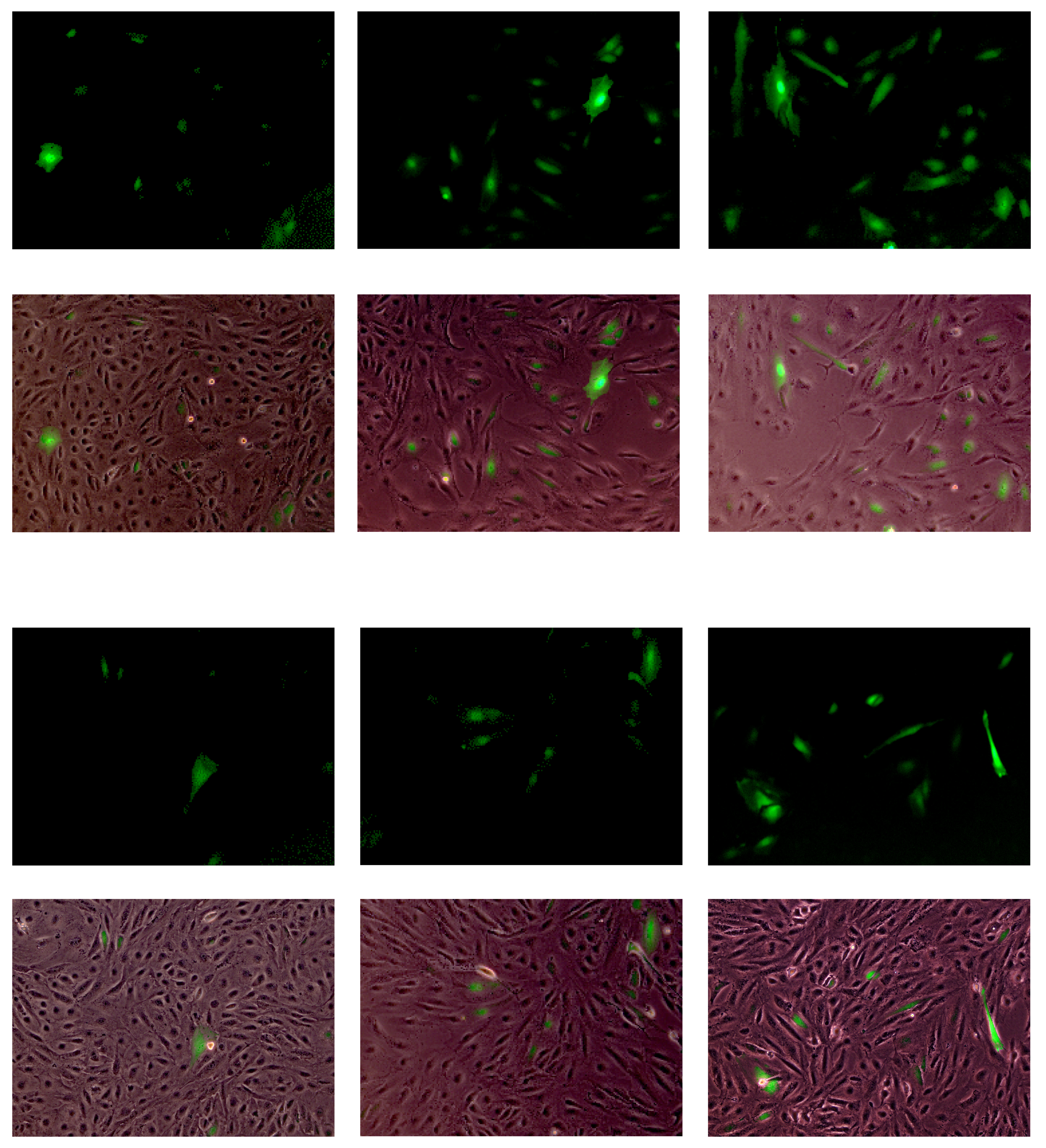

Supplement: Supplementary file 6 — Source data Fig. 4 [file 44319_2024_233_MOESM6_ESM.zip › Figure 4 Source data - zip/Figure 4I.tif]

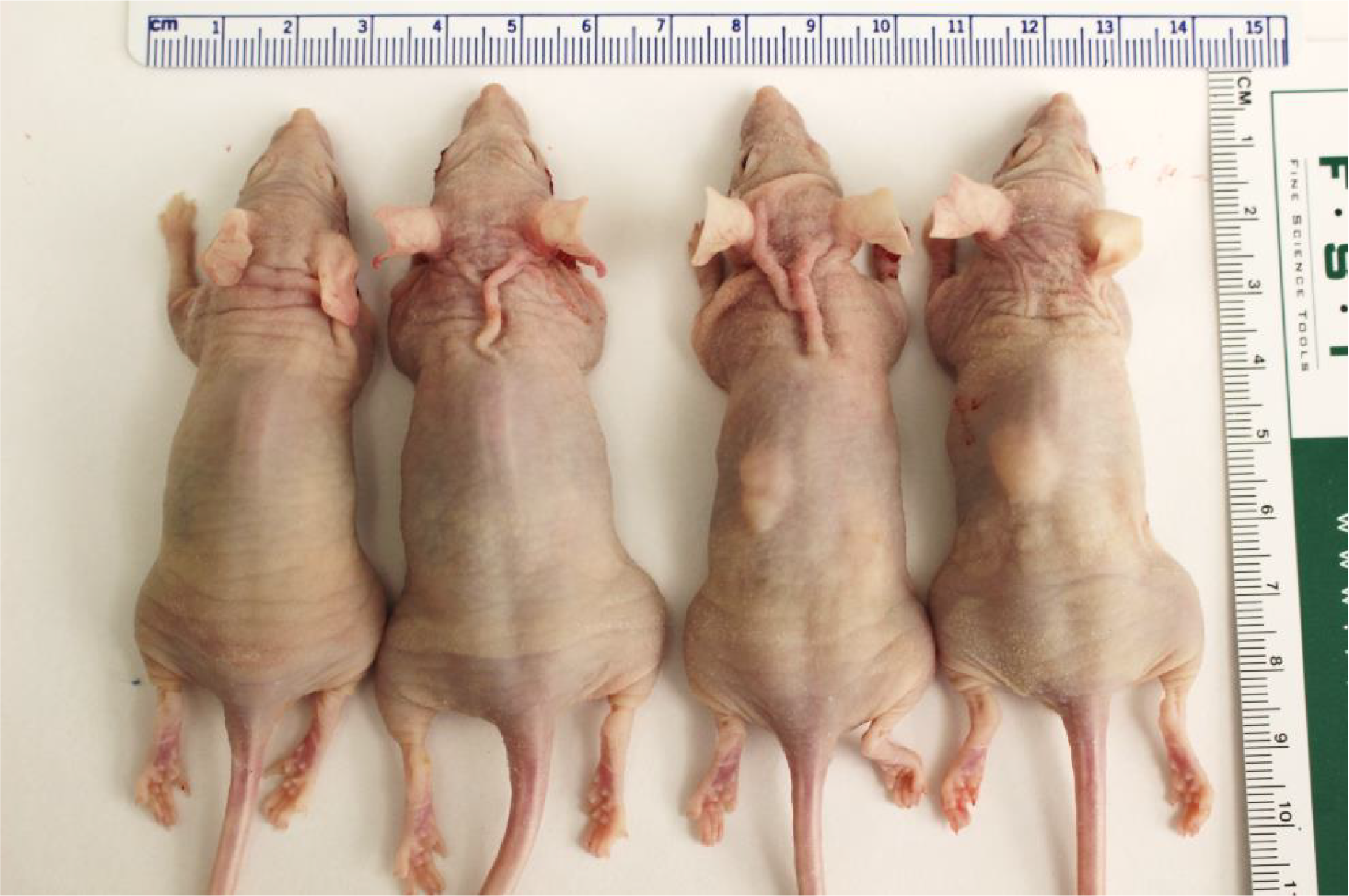

Supplement: Supplementary file 7 — Source data Fig. 5 [file 44319_2024_233_MOESM7_ESM.zip › Figure 5 Source data - zip/Figure 5B.tif]

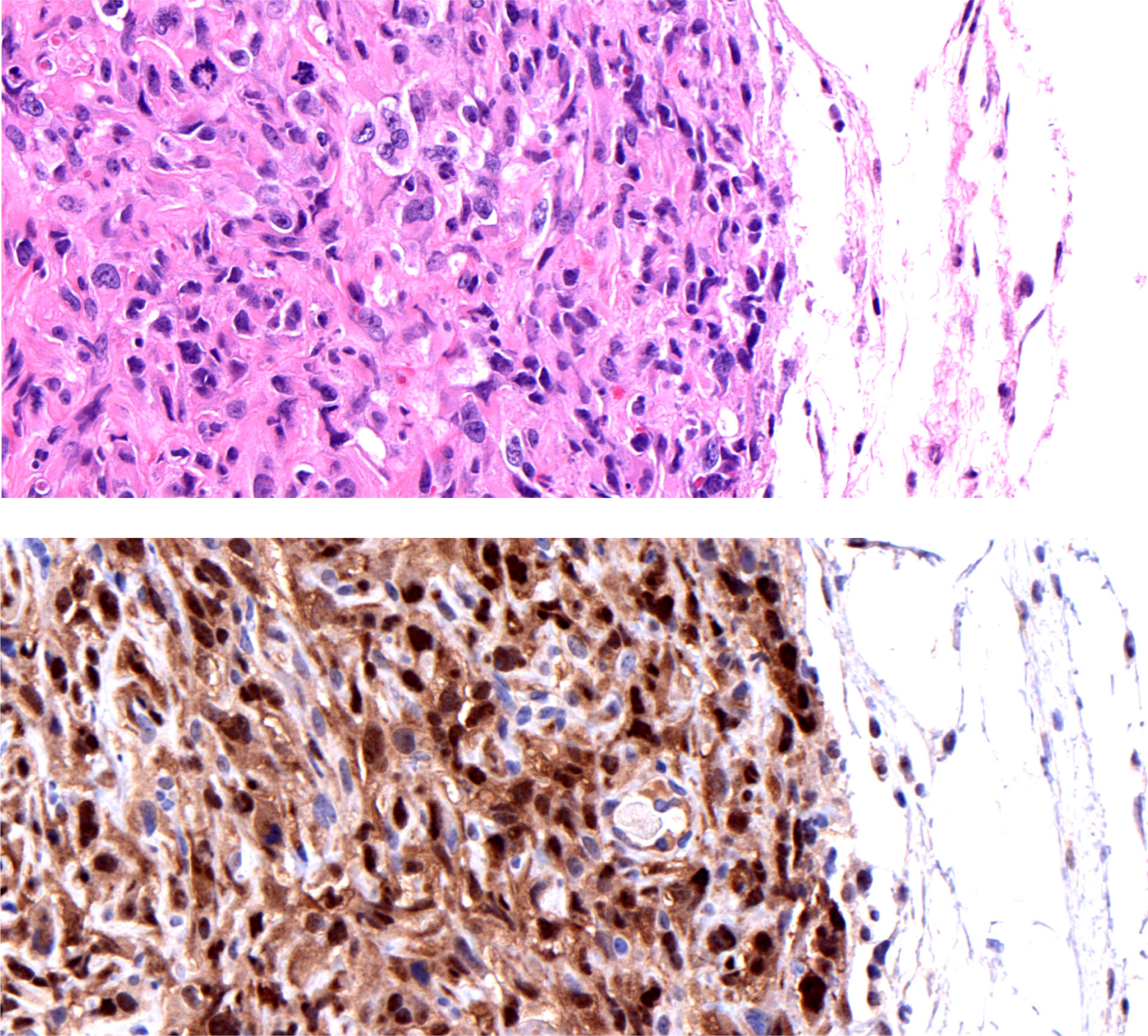

Supplement: Supplementary file 7 — Source data Fig. 5 [file 44319_2024_233_MOESM7_ESM.zip › Figure 5 Source data - zip/Figure 5C&5D.tif]

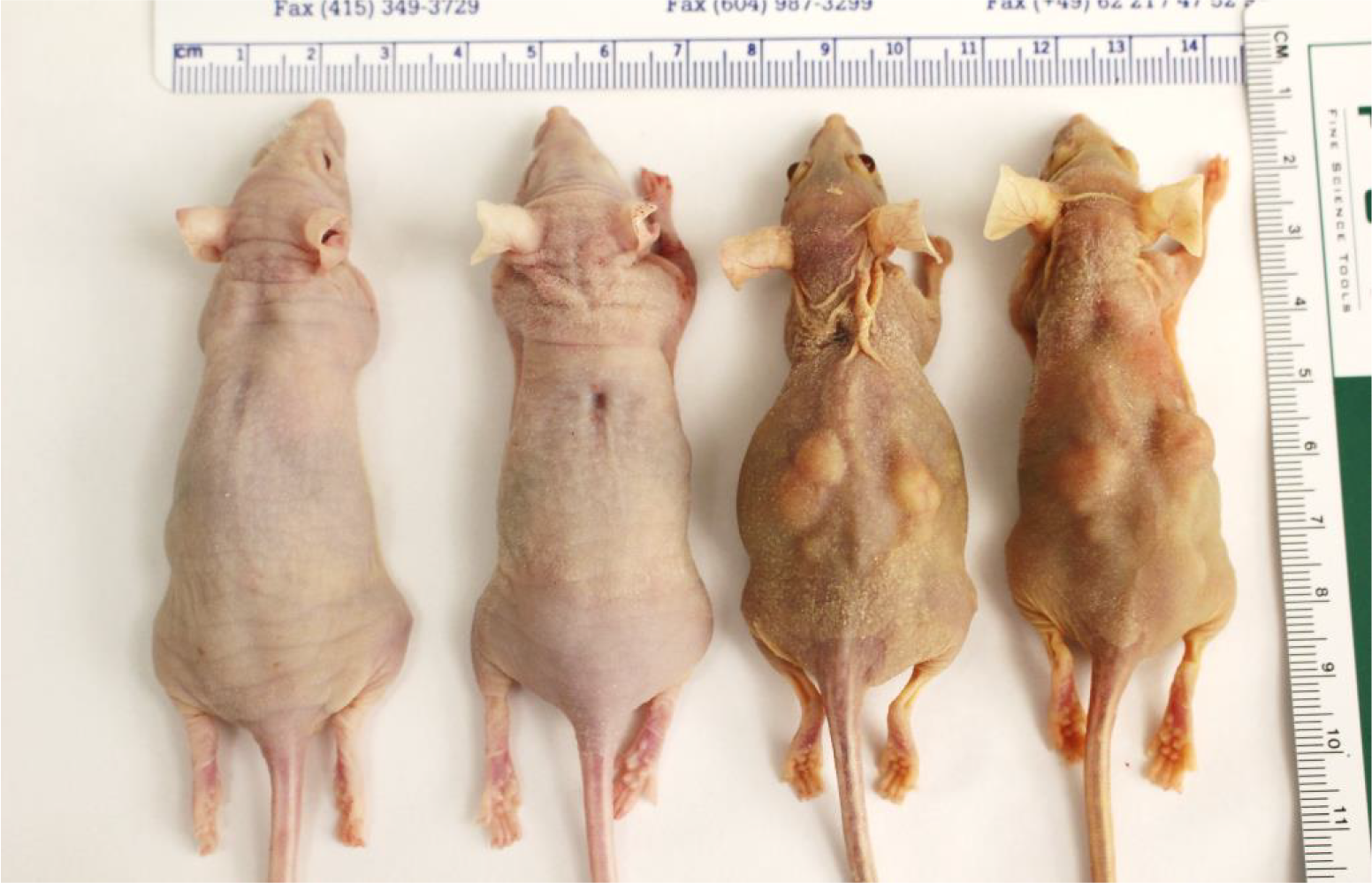

Supplement: Supplementary file 7 — Source data Fig. 5 [file 44319_2024_233_MOESM7_ESM.zip › Figure 5 Source data - zip/Figure 5E.tif]

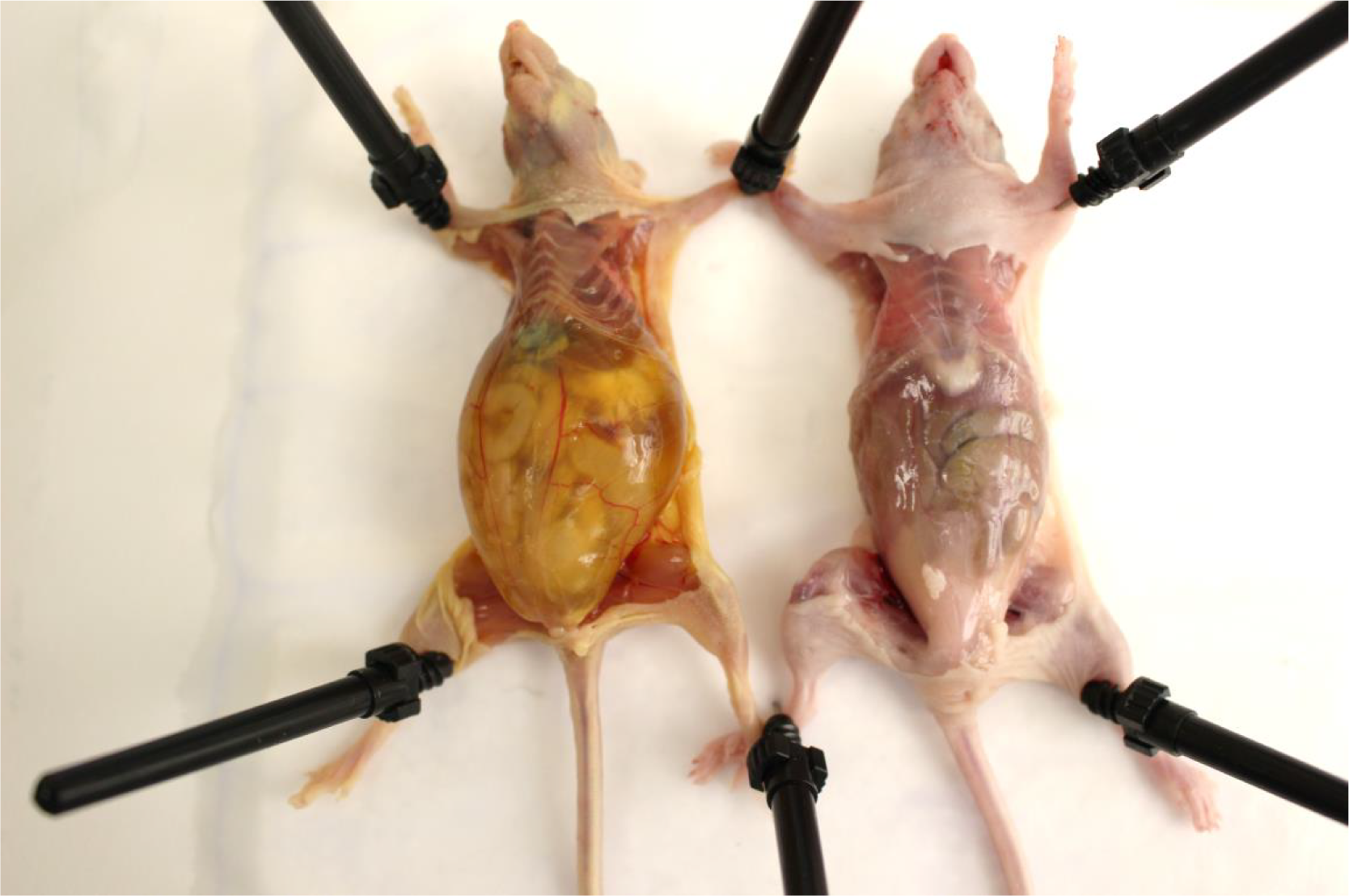

Supplement: Supplementary file 7 — Source data Fig. 5 [file 44319_2024_233_MOESM7_ESM.zip › Figure 5 Source data - zip/Figure 5F.tif]

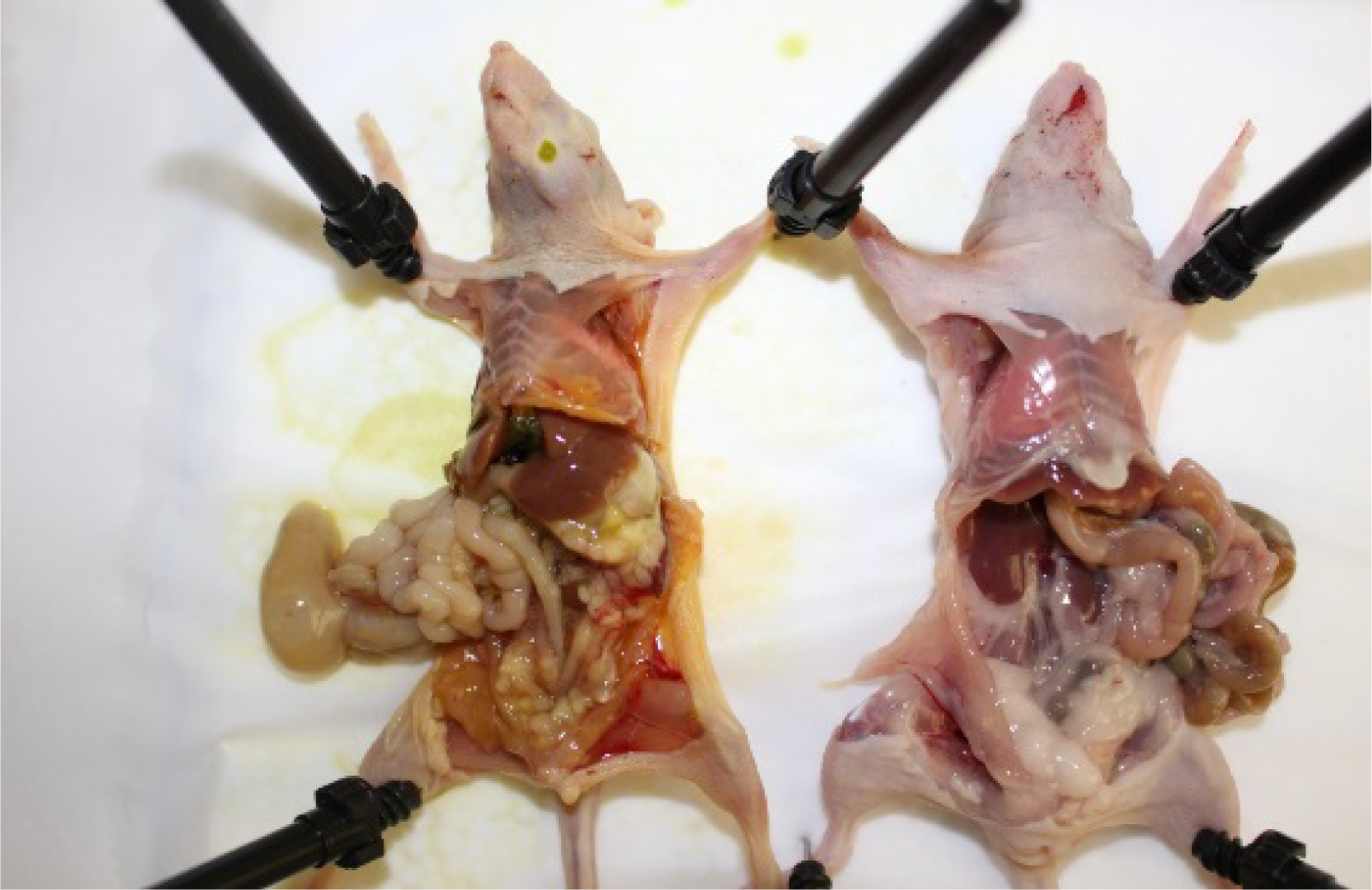

Supplement: Supplementary file 7 — Source data Fig. 5 [file 44319_2024_233_MOESM7_ESM.zip › Figure 5 Source data - zip/Figure 5G.tif]

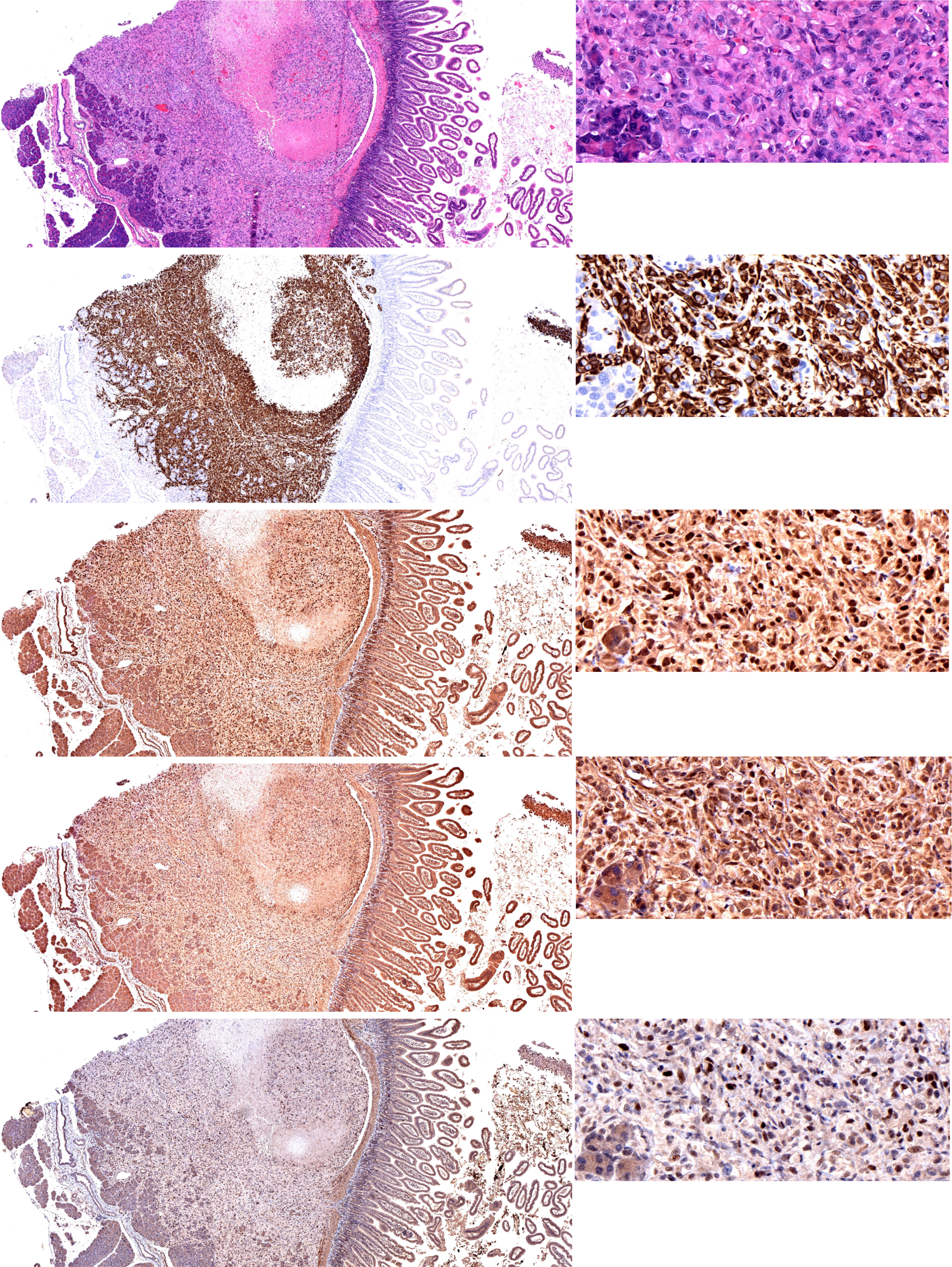

Supplement: Supplementary file 7 — Source data Fig. 5 [file 44319_2024_233_MOESM7_ESM.zip › Figure 5 Source data - zip/Figure 5H-5Q.tif]

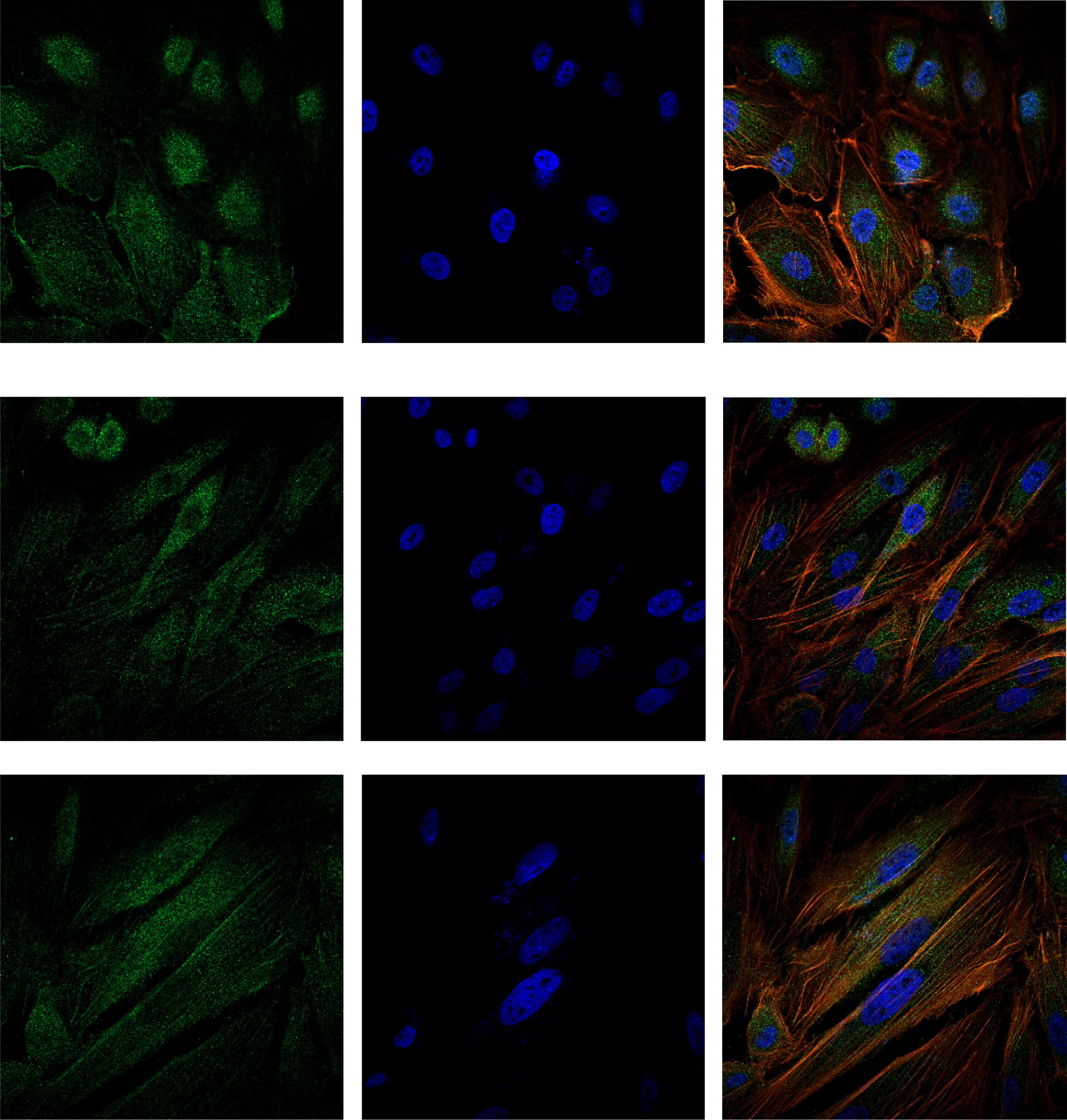

Supplement: Supplementary file 9 — Source data Fig. 7 [file 44319_2024_233_MOESM9_ESM.zip › Figure 7 Source data - zip/Figure 7B-IRF3.tif]

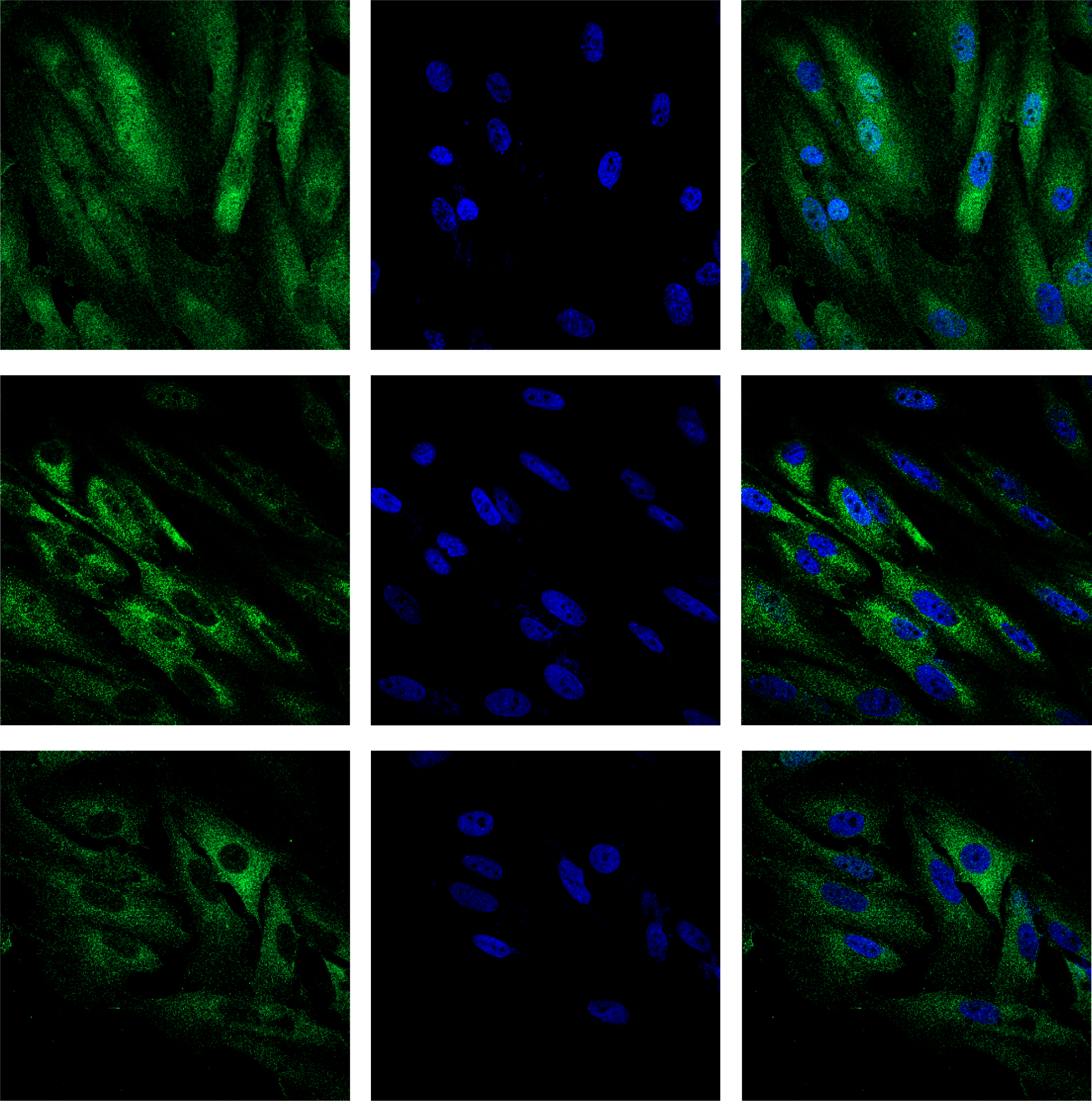

Supplement: Supplementary file 9 — Source data Fig. 7 [file 44319_2024_233_MOESM9_ESM.zip › Figure 7 Source data - zip/Figure 7B-NFKB.tif]

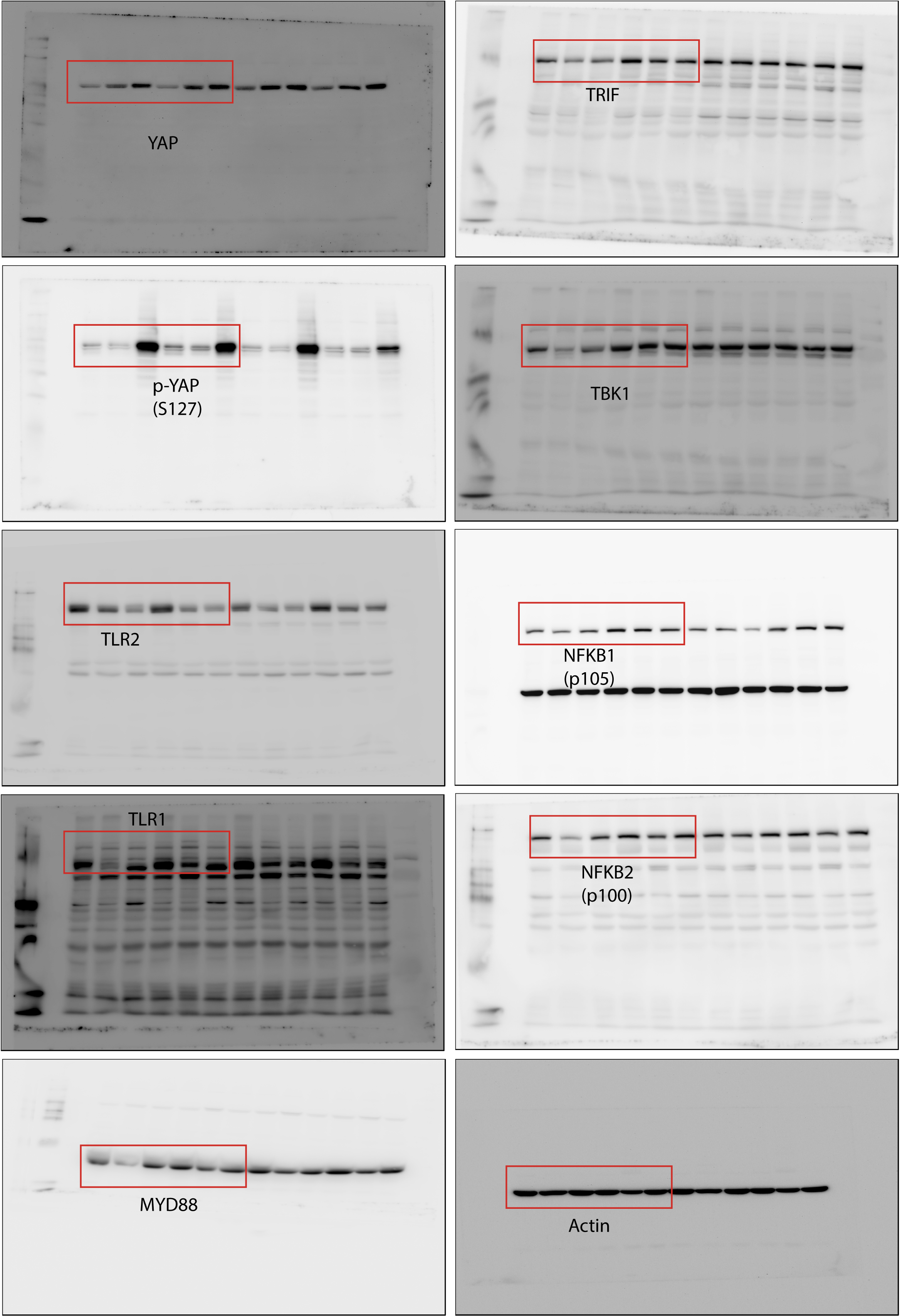

Supplement: Supplementary file 9 — Source data Fig. 7 [file 44319_2024_233_MOESM9_ESM.zip › Figure 7 Source data - zip/Figure 7D.tif]

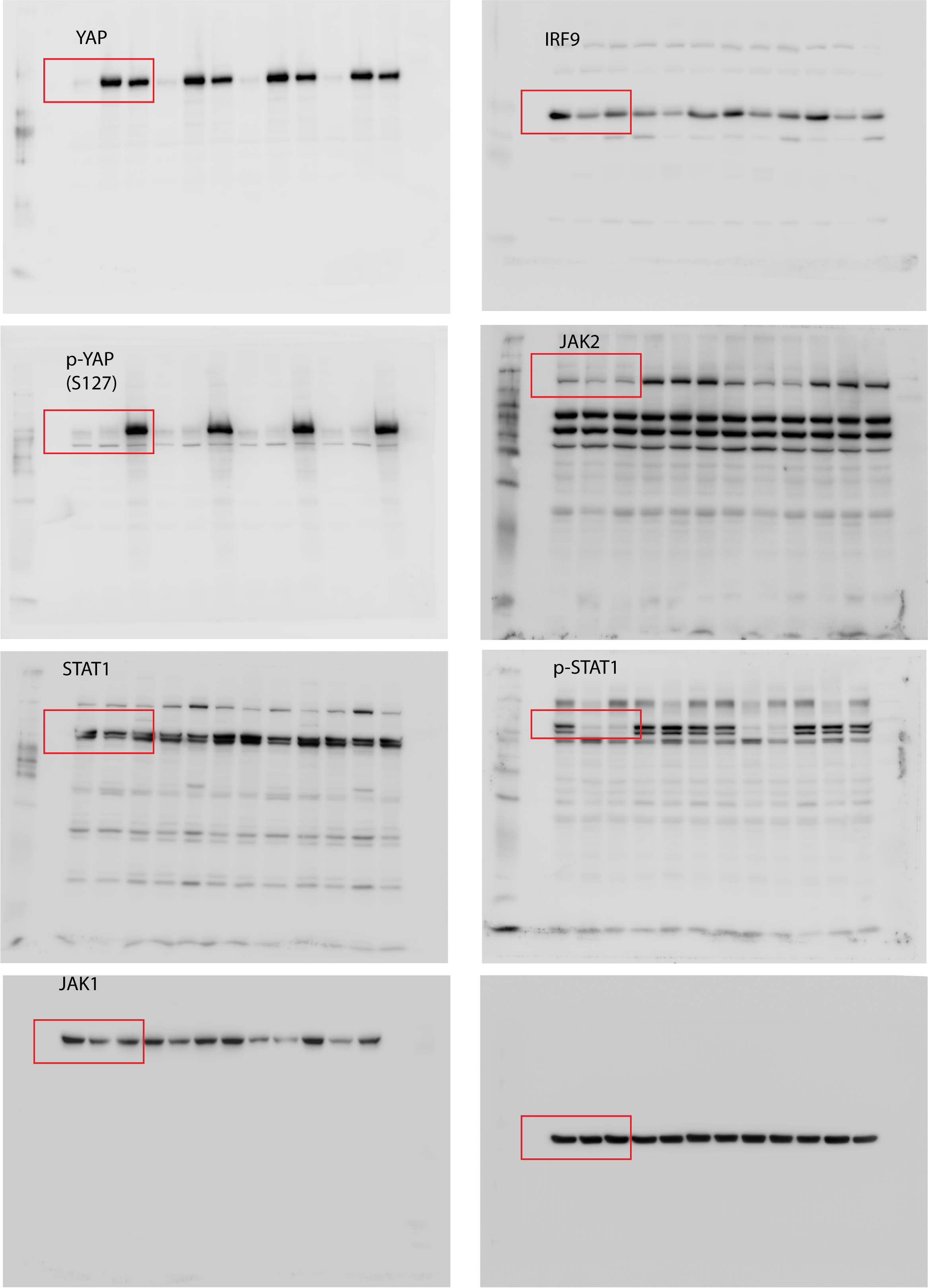

Supplement: Supplementary file 10 — Source data Fig. 8 [file 44319_2024_233_MOESM10_ESM.zip › Figure 8 Source data - zip/Figure 8B.tif]

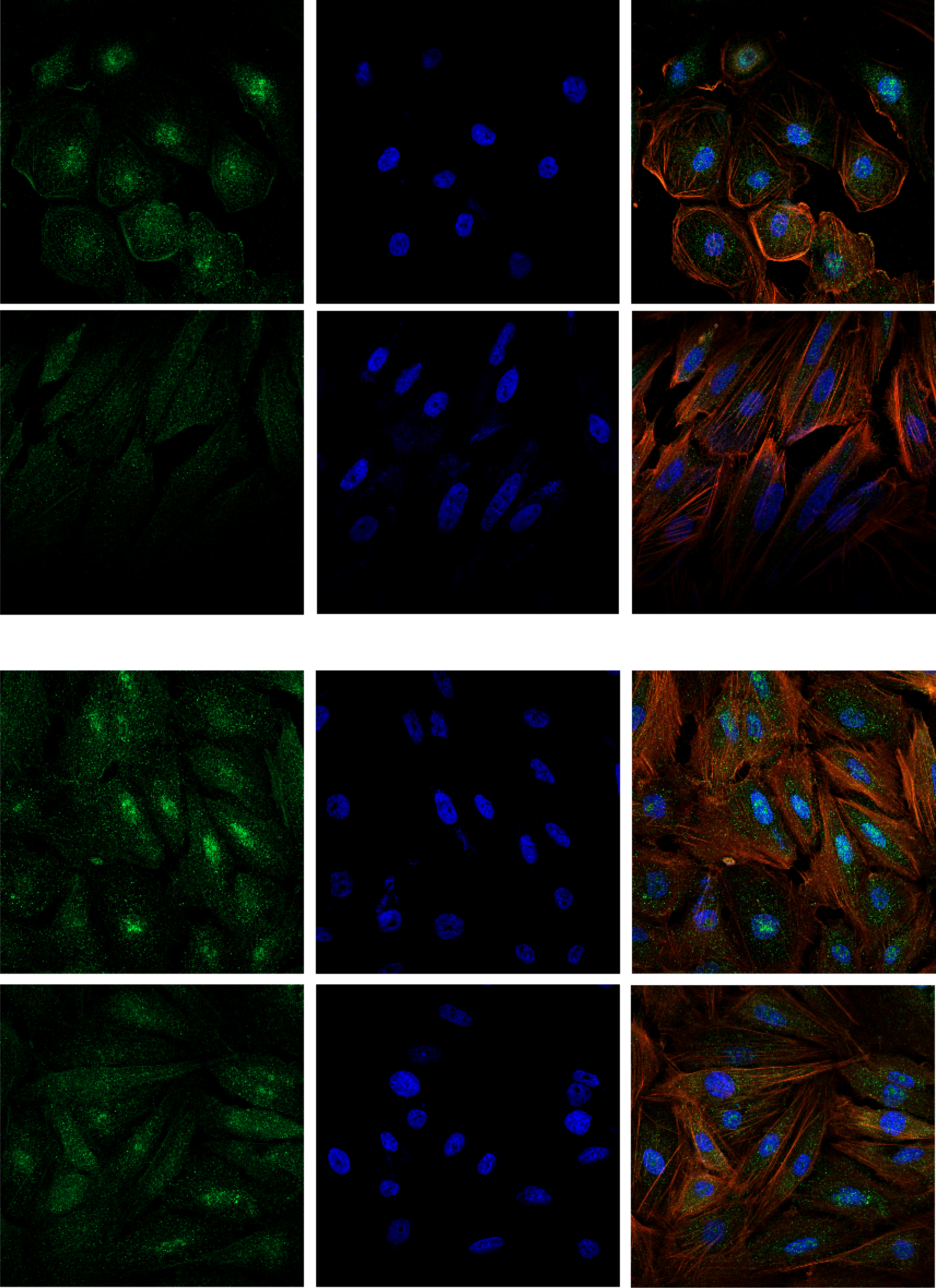

Supplement: Supplementary file 10 — Source data Fig. 8 [file 44319_2024_233_MOESM10_ESM.zip › Figure 8 Source data - zip/Figure 8C-IRF9.tif]

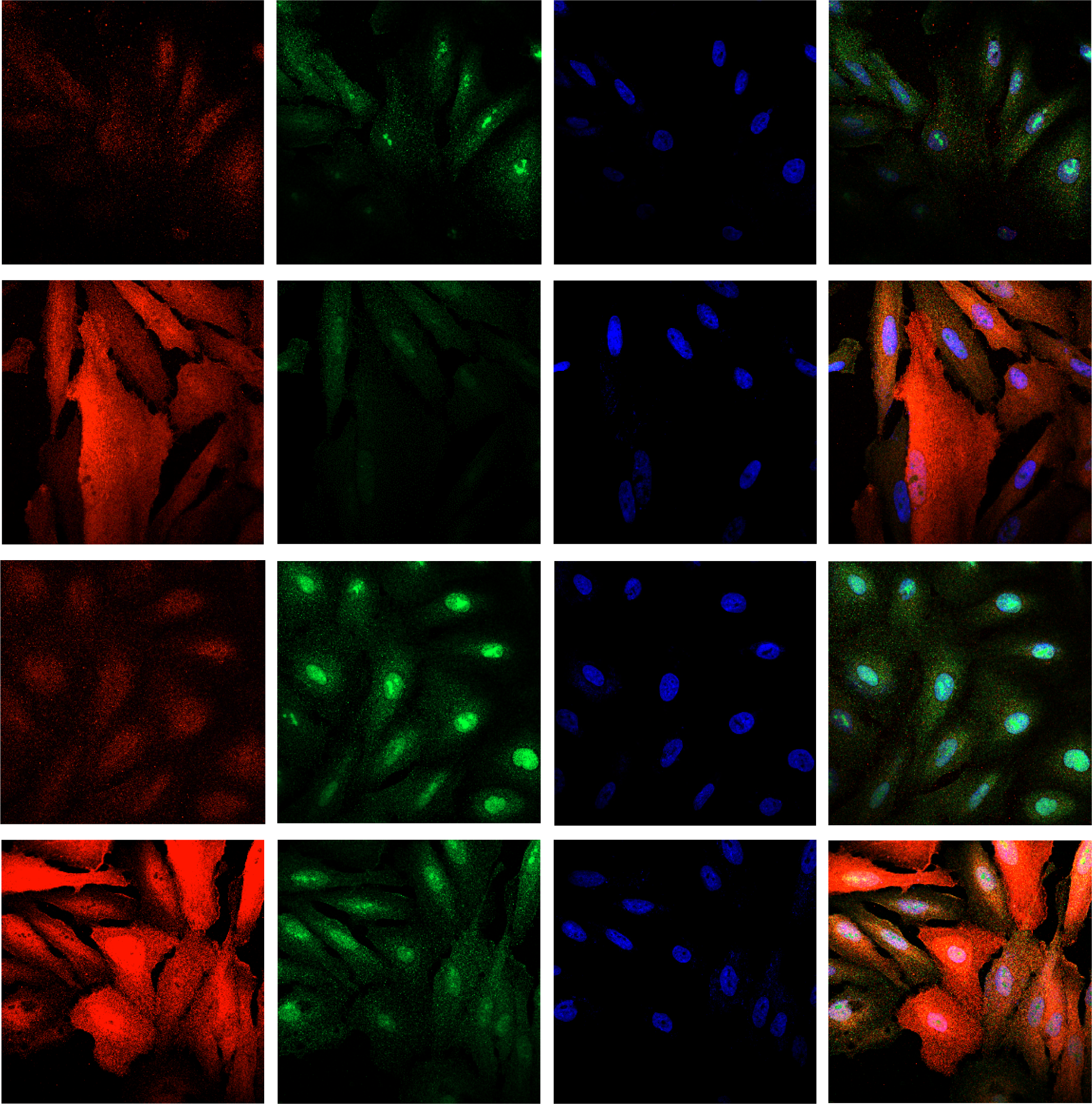

Supplement: Supplementary file 10 — Source data Fig. 8 [file 44319_2024_233_MOESM10_ESM.zip › Figure 8 Source data - zip/Figure 8C-STAT1.tif]

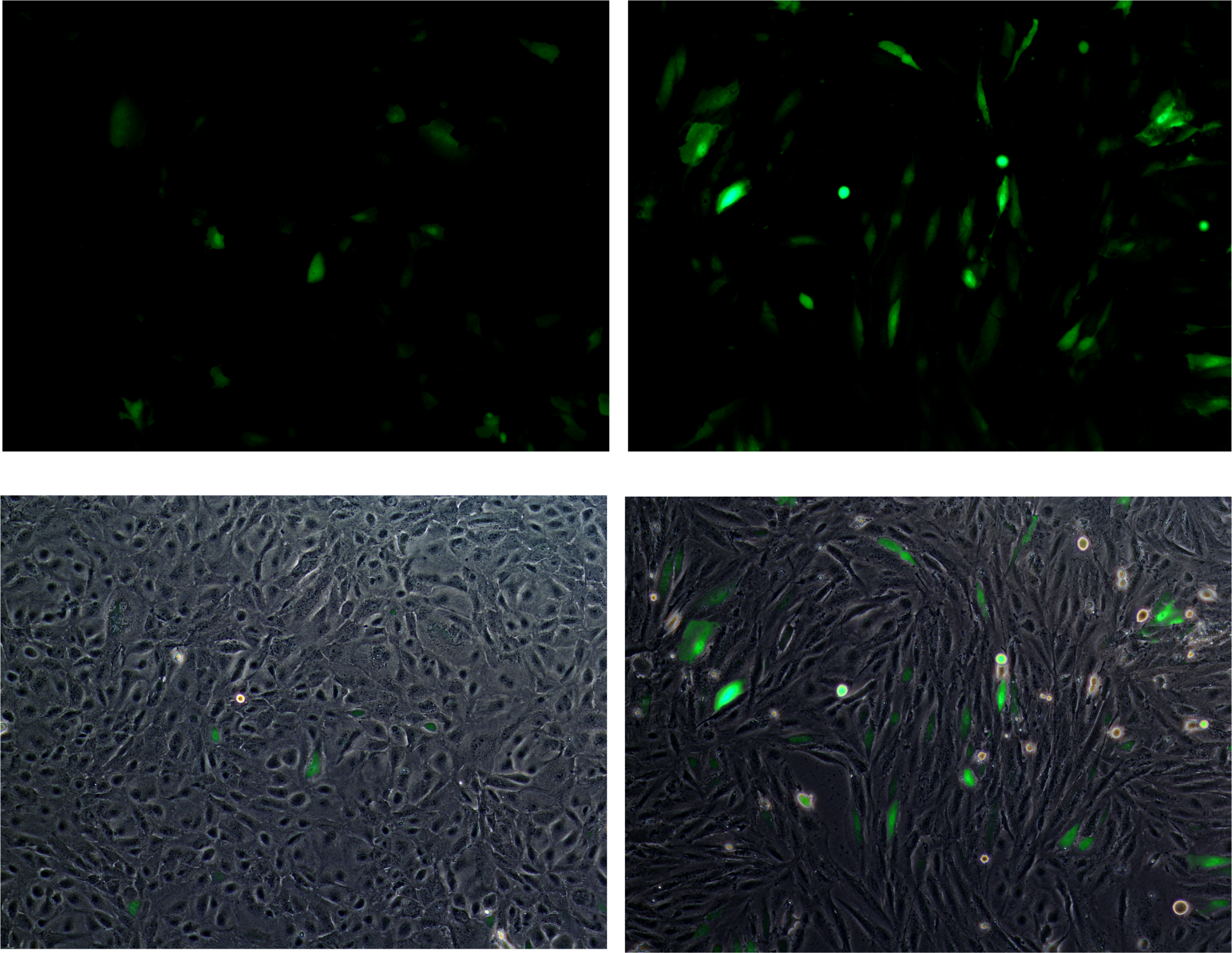

Supplement: Supplementary file 10 — Source data Fig. 8 [file 44319_2024_233_MOESM10_ESM.zip › Figure 8 Source data - zip/Figure 8D-Control.tif]

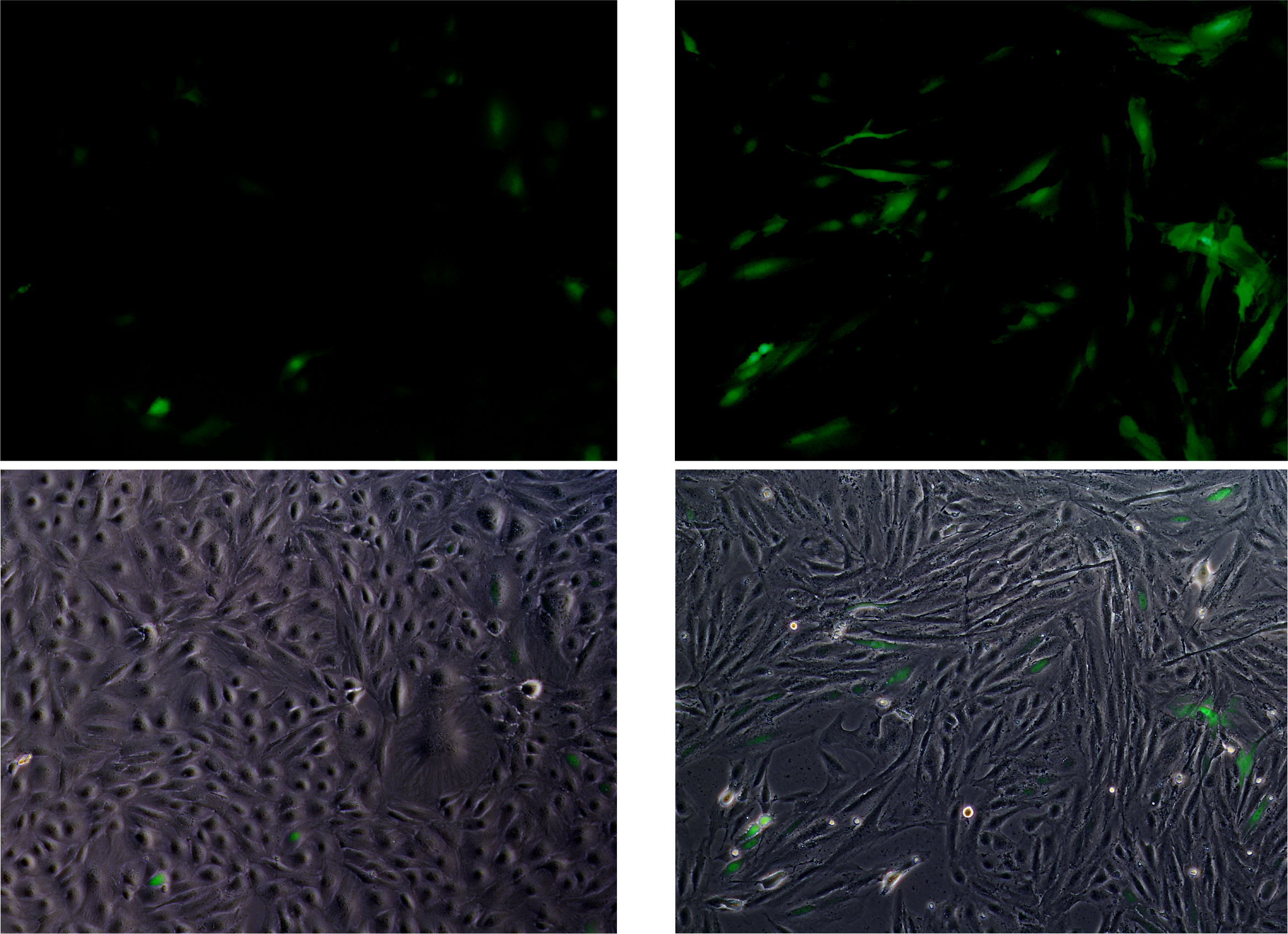

Supplement: Supplementary file 10 — Source data Fig. 8 [file 44319_2024_233_MOESM10_ESM.zip › Figure 8 Source data - zip/Figure 8D-IFNA2.tif]

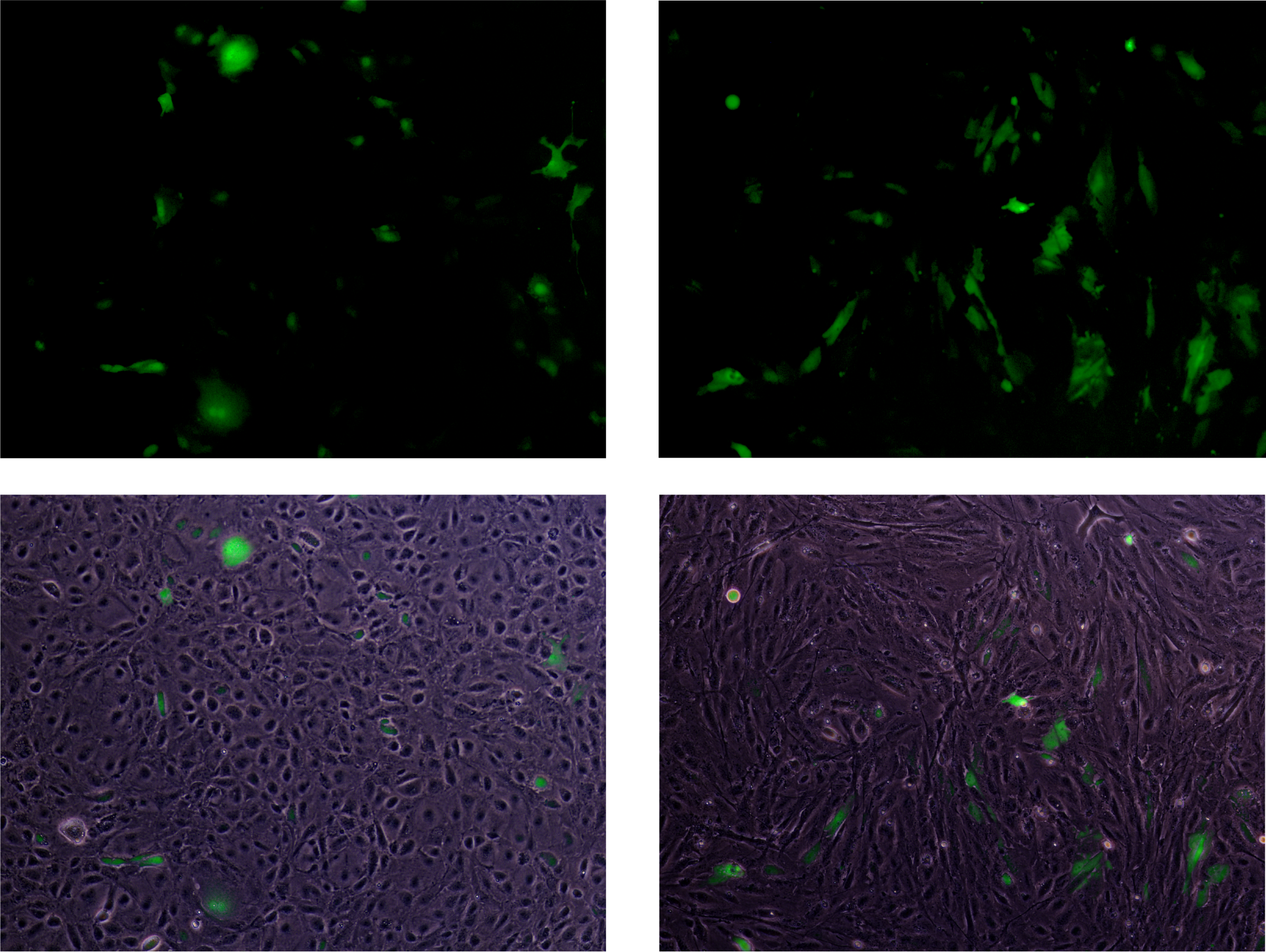

Supplement: Supplementary file 10 — Source data Fig. 8 [file 44319_2024_233_MOESM10_ESM.zip › Figure 8 Source data - zip/Figure 8D-Ruxolitinib.tif]

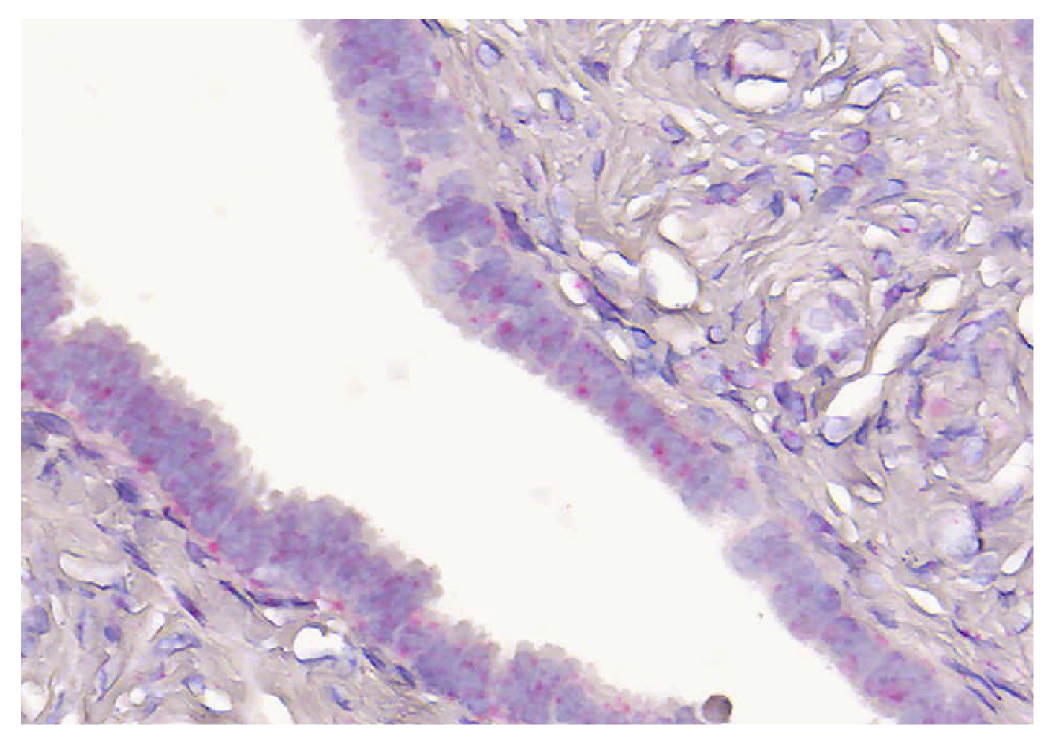

Supplement: Supplementary file 11 — EV Figures Source Data [file 44319_2024_233_MOESM11_ESM.zip › Figure EV1 source data - zip/Figure EV1A-01.tif]

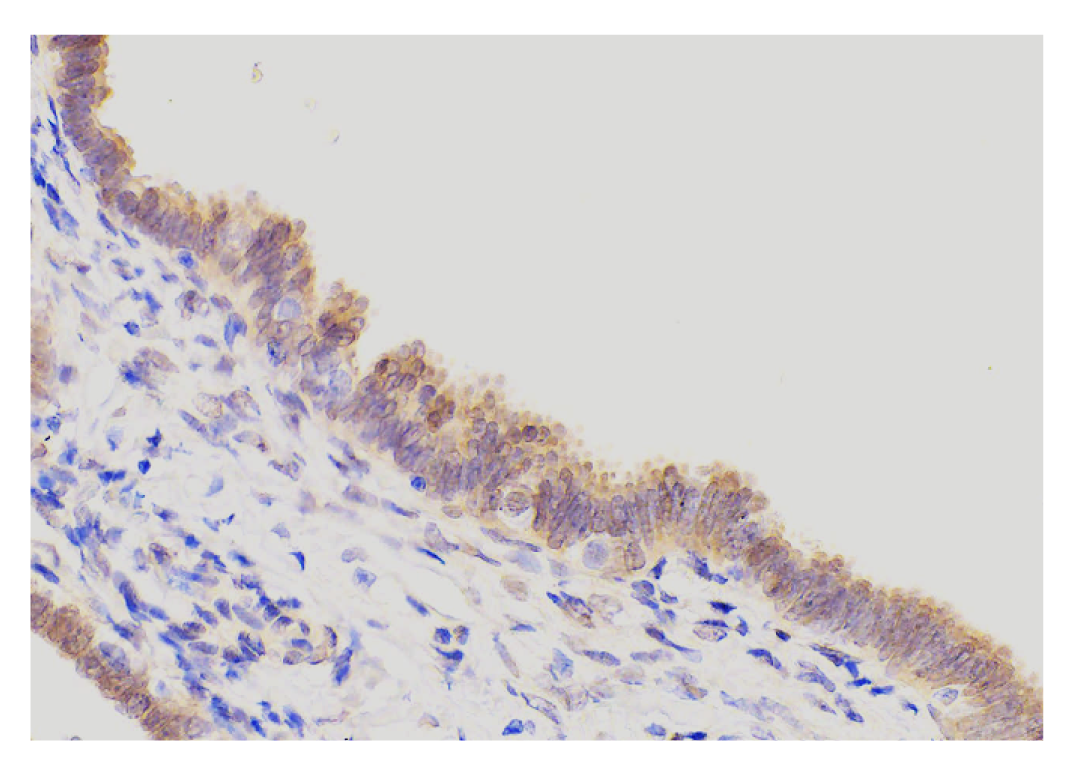

Supplement: Supplementary file 11 — EV Figures Source Data [file 44319_2024_233_MOESM11_ESM.zip › Figure EV1 source data - zip/Figure EV1B-01.tif]

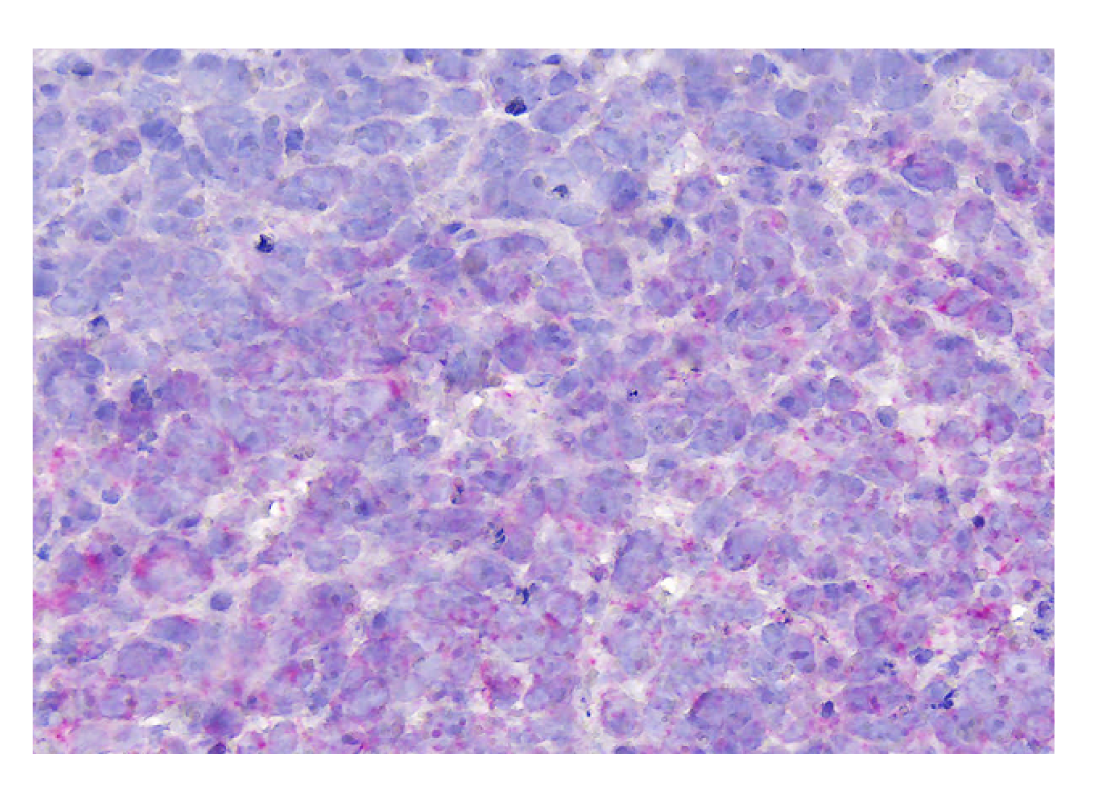

Supplement: Supplementary file 11 — EV Figures Source Data [file 44319_2024_233_MOESM11_ESM.zip › Figure EV1 source data - zip/Figure EV1C-01.tif]

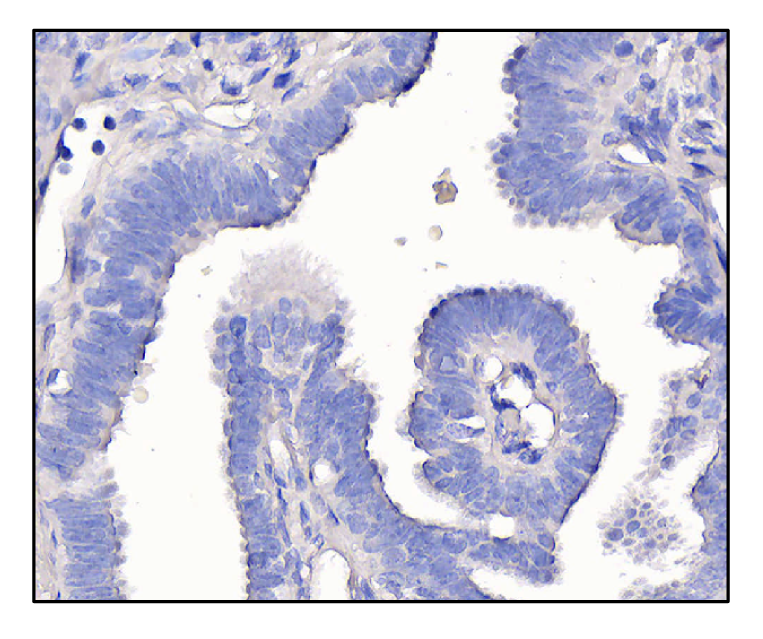

Supplement: Supplementary file 11 — EV Figures Source Data [file 44319_2024_233_MOESM11_ESM.zip › Figure EV1 source data - zip/Figure EV1D - STIC negative-01.tif]

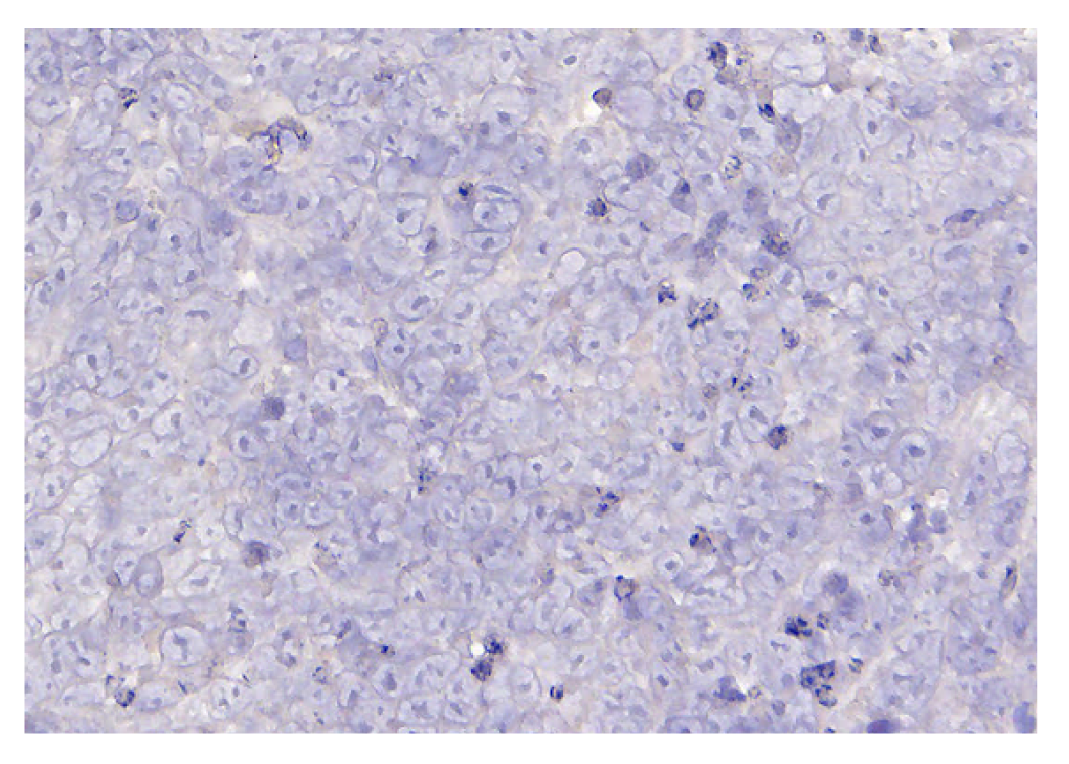

Supplement: Supplementary file 11 — EV Figures Source Data [file 44319_2024_233_MOESM11_ESM.zip › Figure EV1 source data - zip/Figure EV1D - Xenograft positive-01.tif]

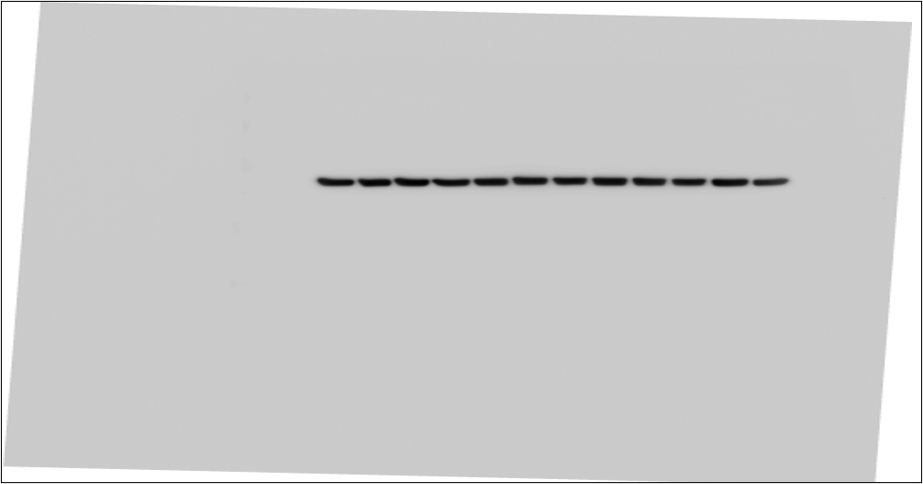

Supplement: Supplementary file 11 — EV Figures Source Data [file 44319_2024_233_MOESM11_ESM.zip › Figure EV3 source data - zip/Fig EV3A - beta-Actin.tif]

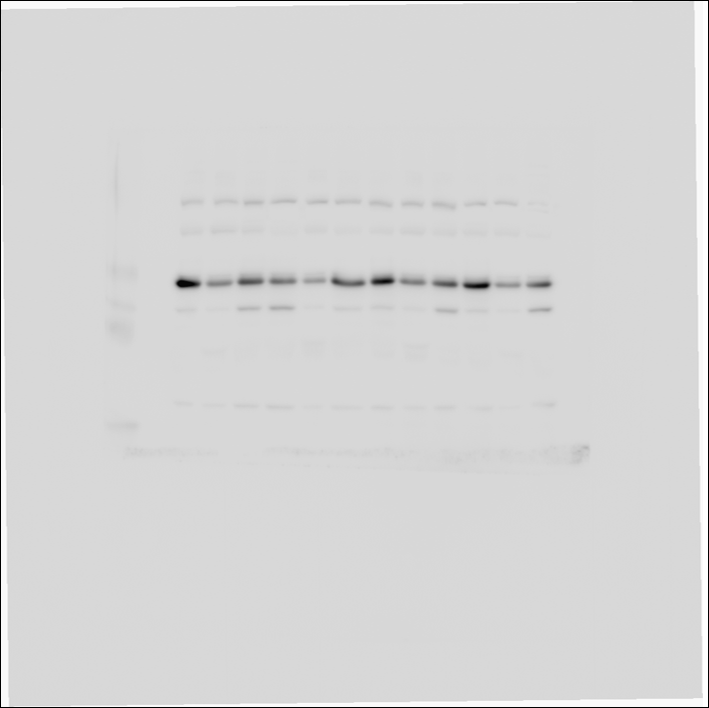

Supplement: Supplementary file 11 — EV Figures Source Data [file 44319_2024_233_MOESM11_ESM.zip › Figure EV3 source data - zip/Fig EV3A - IRF9.tif]

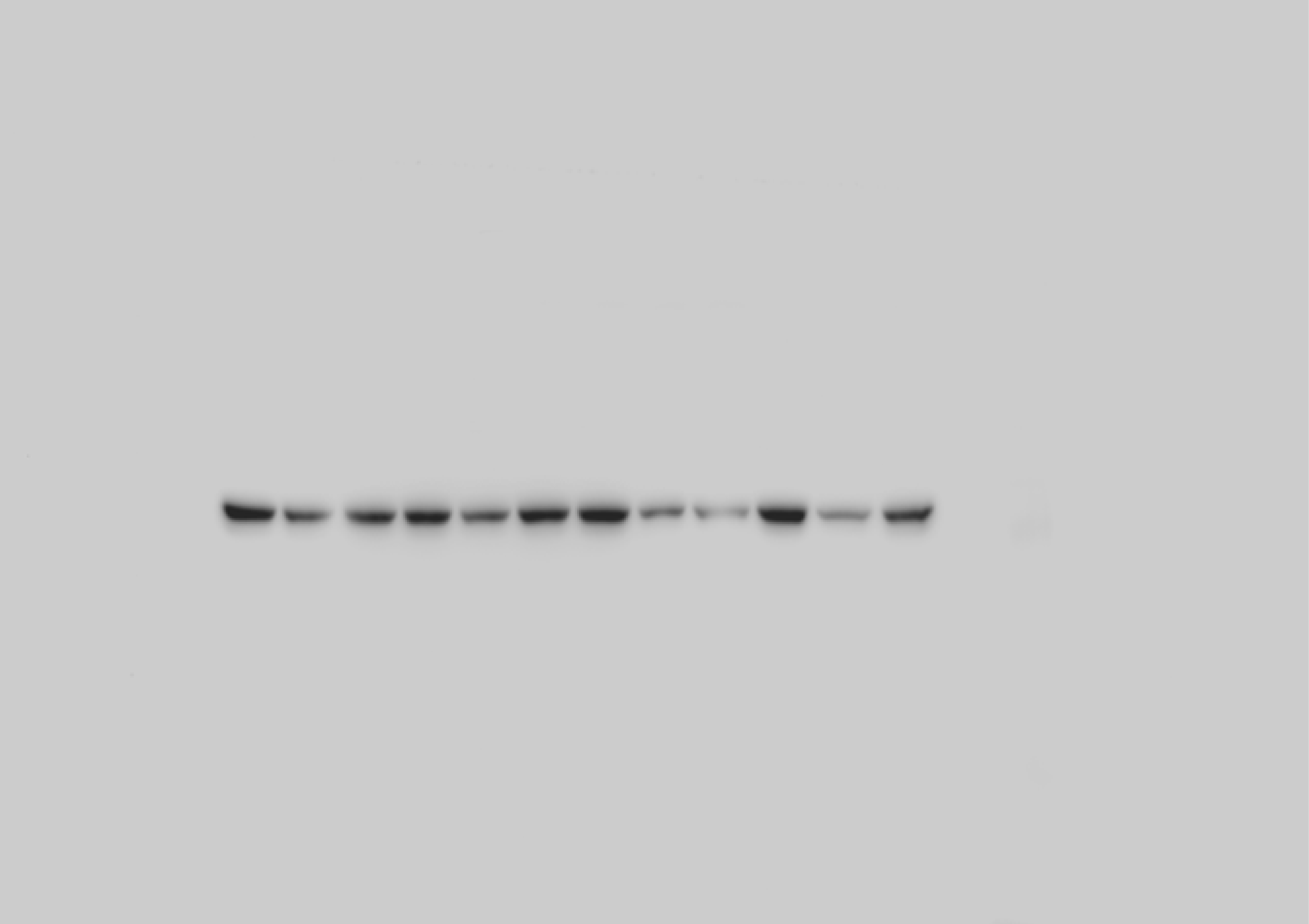

Supplement: Supplementary file 11 — EV Figures Source Data [file 44319_2024_233_MOESM11_ESM.zip › Figure EV3 source data - zip/Fig EV3A - JAK1.tif]

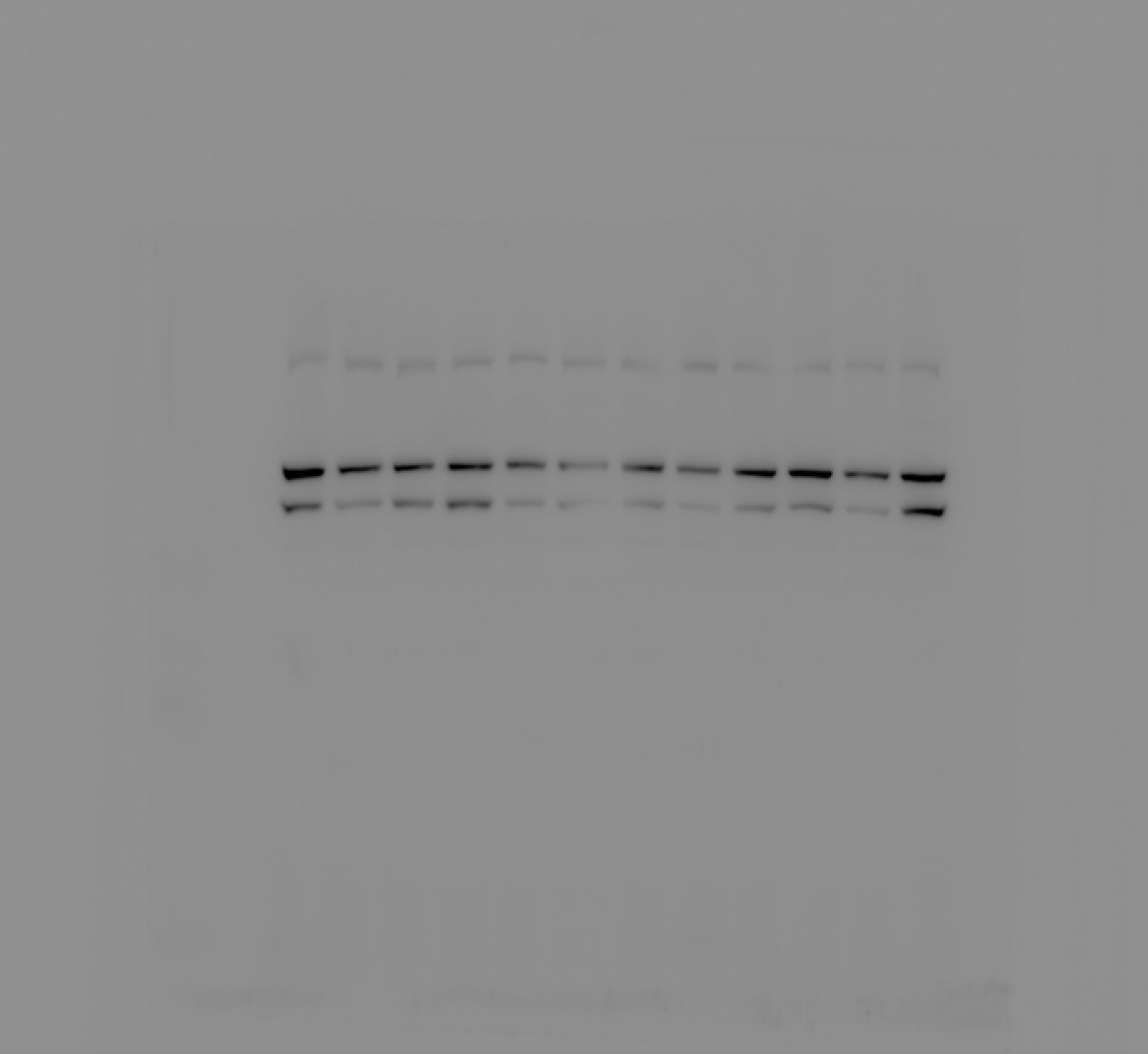

Supplement: Supplementary file 11 — EV Figures Source Data [file 44319_2024_233_MOESM11_ESM.zip › Figure EV3 source data - zip/Fig EV3A - JAK2.tif]

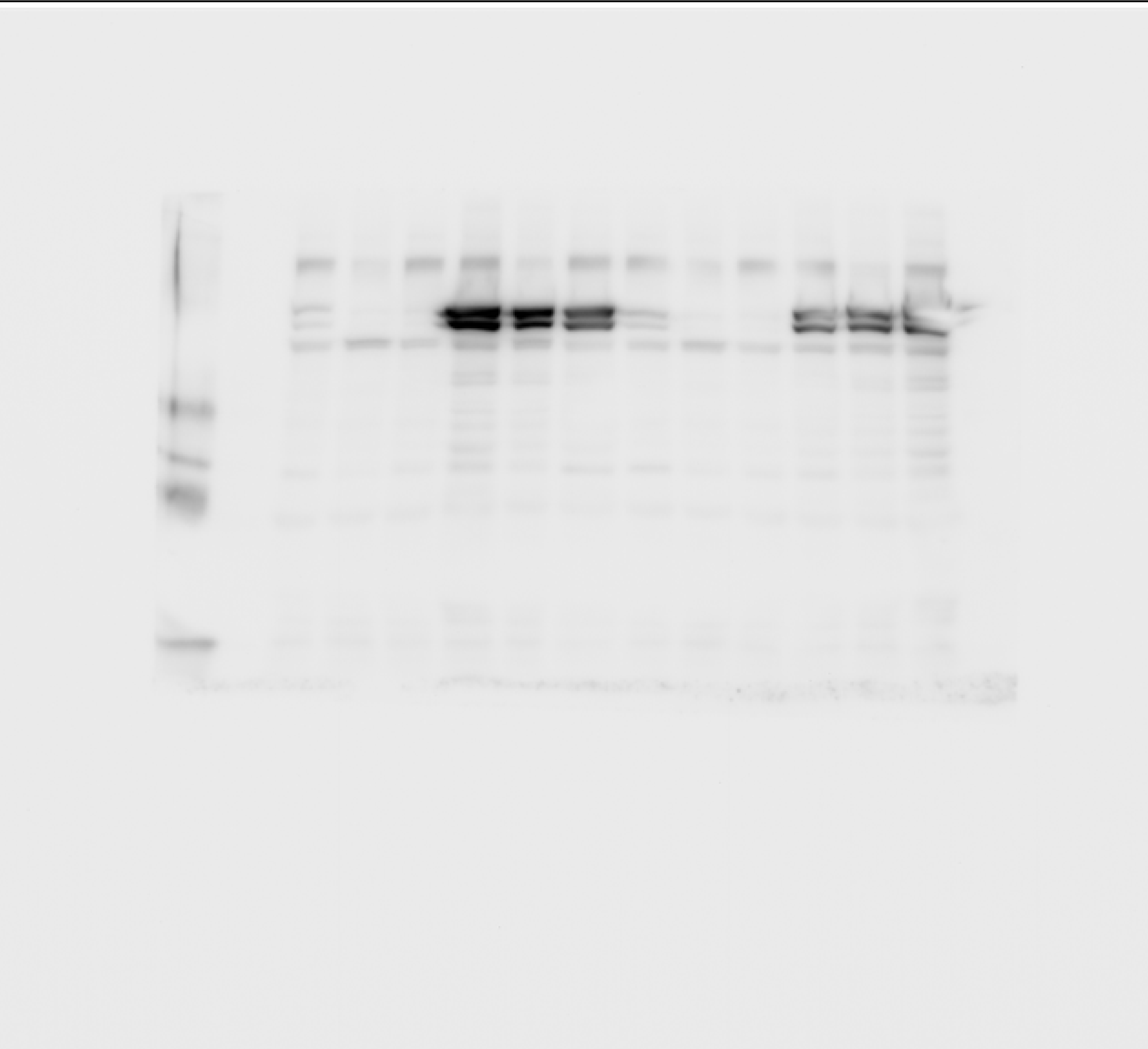

Supplement: Supplementary file 11 — EV Figures Source Data [file 44319_2024_233_MOESM11_ESM.zip › Figure EV3 source data - zip/Fig EV3A - p-STAT1.tif]

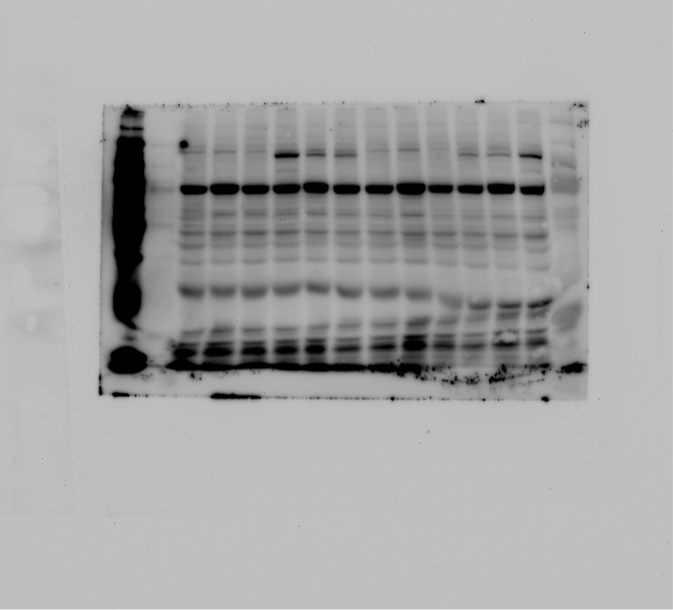

Supplement: Supplementary file 11 — EV Figures Source Data [file 44319_2024_233_MOESM11_ESM.zip › Figure EV3 source data - zip/Fig EV3A - p-STAT2.tif]

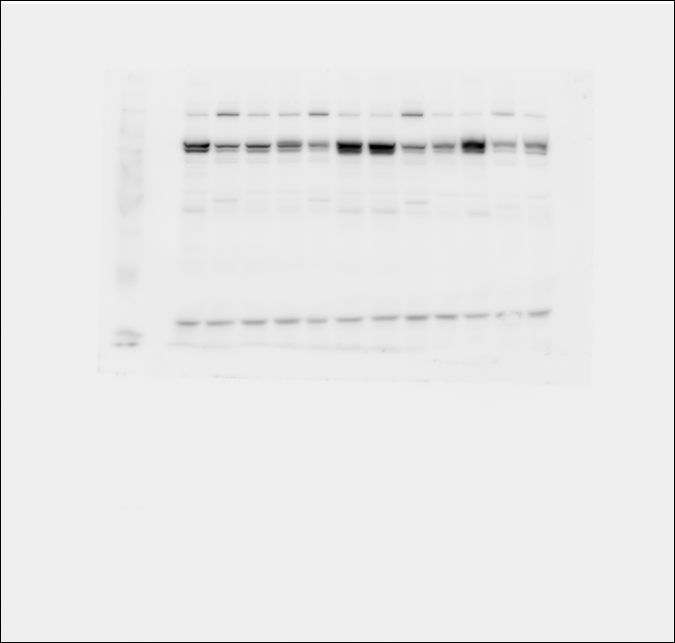

Supplement: Supplementary file 11 — EV Figures Source Data [file 44319_2024_233_MOESM11_ESM.zip › Figure EV3 source data - zip/Fig EV3A - STAT1.tif]

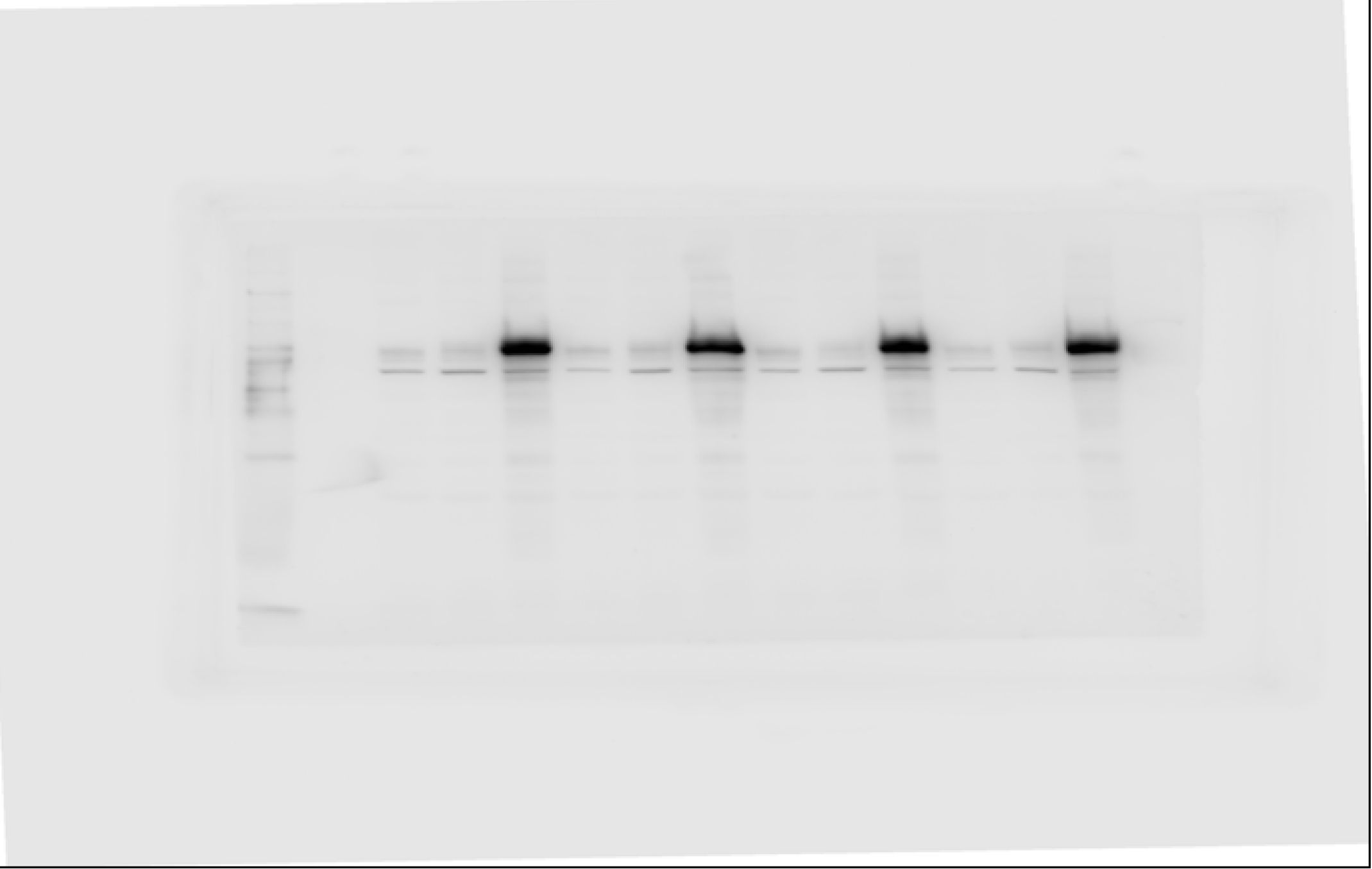

Supplement: Supplementary file 11 — EV Figures Source Data [file 44319_2024_233_MOESM11_ESM.zip › Figure EV3 source data - zip/Fig EV3A - YAP S127A.tif]

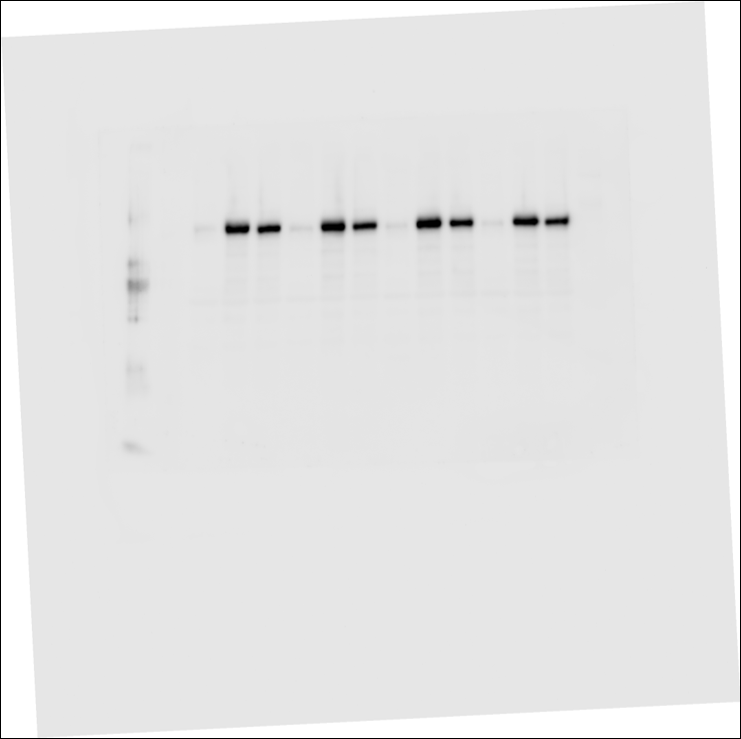

Supplement: Supplementary file 11 — EV Figures Source Data [file 44319_2024_233_MOESM11_ESM.zip › Figure EV3 source data - zip/Fig EV3A - YAP.tif]
