# Supplementary material for: HPV-YAP1 oncogenic alliance drives malignant transformation of fallopian tube epithelial cells
Source: EMBO Rep. 2024 Sep 13;25(10):26. doi: 10.1038/s44319-024-00233-3 (PMC11467260; doi:10.1038/s44319-024-00233-3)
Supplement: Supplementary file 8 — Source data Fig. 6 [file 44319_2024_233_MOESM8_ESM.zip › Figure 6 Source data - zip/Figure 6B 6C 6D source data.docx]

The datasets produced in Figure 6 are available in the following databases:

- RNA-Seq data: Gene Expression Omnibus accession GSE268836:

( <https://www.ncbi.nlm.nih.gov/geo/query/acc.cgi?acc=GSE268836>)
