# Supplementary material for: HPV-YAP1 oncogenic alliance drives malignant transformation of fallopian tube epithelial cells
Source: EMBO Rep. 2024 Sep 13;25(10):26. doi: 10.1038/s44319-024-00233-3 (PMC11467260; doi:10.1038/s44319-024-00233-3)
Supplement: Supplementary file 12 — Expanded View Figures [file 44319_2024_233_MOESM12_ESM.pdf]

Expanded View Figures

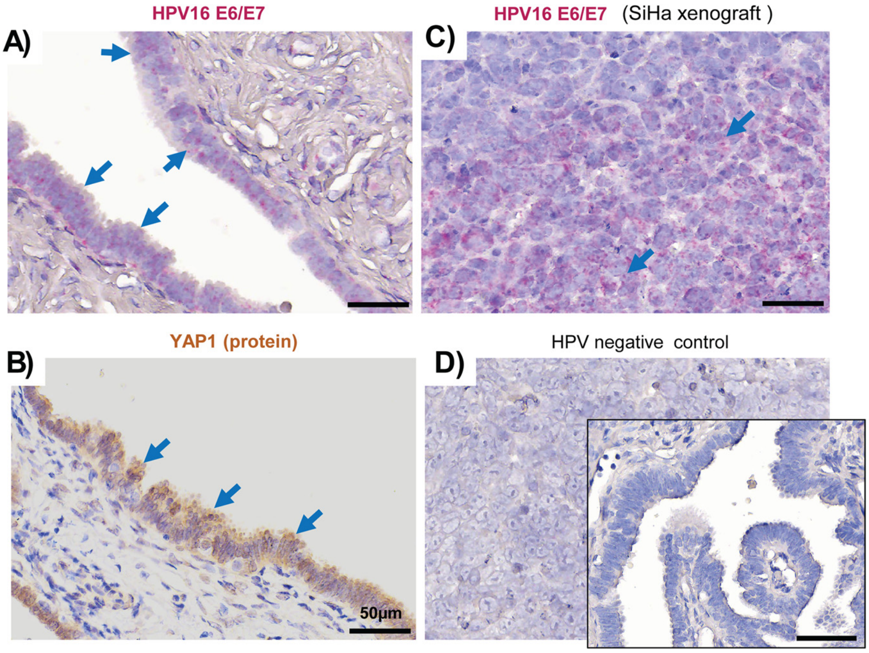

Figure EV1. Expression of HPV16 E6/E7 in the Fallopian tube STIC lesion (precursor of HGSOC) detected by RNA scope.

(**A**) A representative image showing the expression of HPV16 E6/E7 mRNA (in pink) in fallopian tube STIC lesion (arrow) of a human patient (sample-GU980150-E10). E6/E7 were detected and visualized using the RNA scope technique. Scale bar: 50 µm. (**B**) A representative image showing the expression of YAP1 protein (in brown) in fallopian tube STIC lesion. YAP1 protein was detected and visualized by immunohistochemistry. Arrows point to neoplastic growth of epithelial cells with nuclear YAP1 protein. Scale bar: 50 µm. (**C**) A representative image showing the expression of HPV16 E6/E7 mRNA (in pink) in SiHa cell xenograft tumor tissues (positive control). Blue arrows point to the HPV16 E6/E7 positive cells (in pink). Scale bar: 50 µm. (**D**) Representative images showing negative staining (non-targeting probe) of HPV16 E6/E7 mRNA in SiHa cell xenograft tumor tissues and human STIC lesion (insert). Scale bar: 50 µm. Source data are available online for this figure.

 © The Author(s)

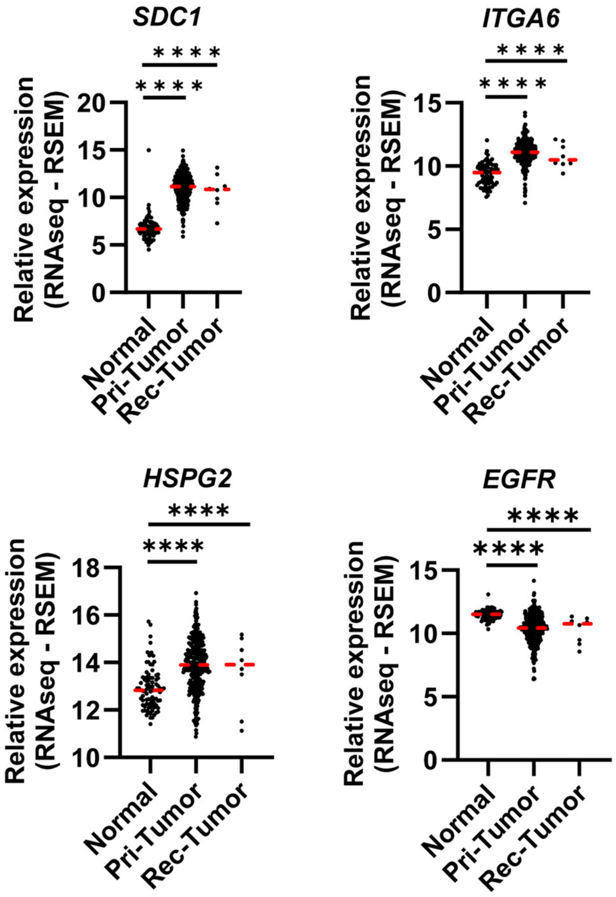

Figure EV2. Expression of genes encoding the putative HPV receptor molecules in normal ovarian tissues, primary ovarian tumors, and recurrent ovarian tumor tissues.

The TCGA TARGET GTEx study online tool (https://xenabrowser.net/) was used to compare the expression of genes encoding the putative HPV receptor molecules in normal ovarian tissues ($n = 88$ normal human samples), primary ovarian tumors ($n = 418$ patient samples), and recurrent ovarian tumor tissues ($n = 8$ patient samples). Data were taken from the UCSC RNA-seq Compendium, where TCGA, TARGET, and GTEx samples are re-analyzed using the same RNA-seq pipeline. Extracted data were analyzed for significance using the one-way ANOVA followed by the Tukey's post hoc test. A value of $P < 0.05$ was considered statistically significant. ****$P < 0.0001$, compared to the normal control group (Normal). Exact *P* values for each gene are presented with the source data of this figure, which is available online. Source data are available online for this figure.

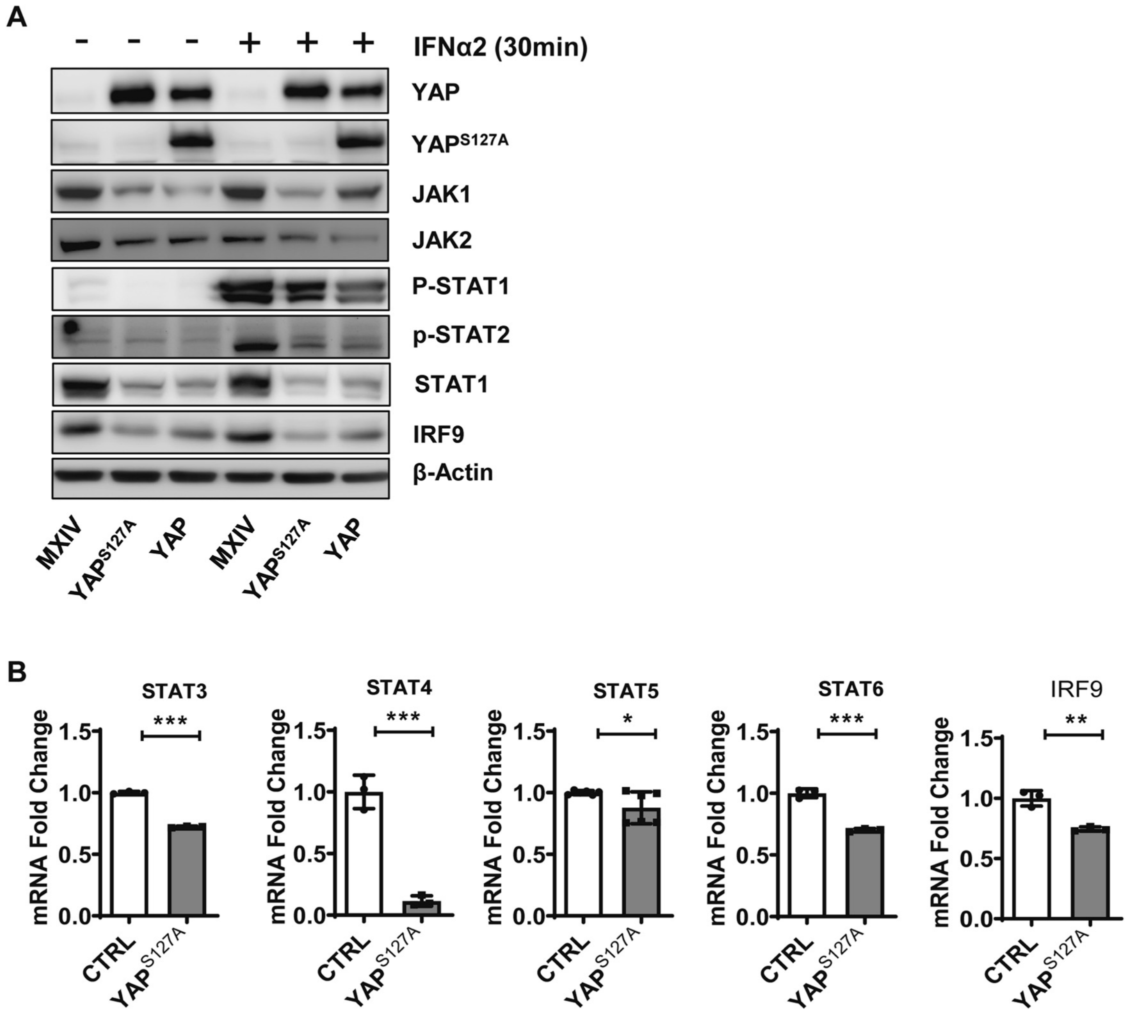

Figure EV3. Constitutive activation of YAP inhibits the type I interferon (JAK/STAT) signaling pathway in fallopian tube secretory epithelial cells (FNE1).

(**A**) Representative blots showing expression and activation of major components of the JAK/STAT/IRF9 pathway in FNE1-MX (control), FNE1-YAP, and FNE1-YAP^{S127} cells with or without IFNα2b treatment for 30 min. Ectopic expression of YAP or YAP^{S127A} in FNE1 cells suppressed IFNα2-induced phosphorylation of STAT1/2. (**B**) Quantitative data showing that transcription of STATs are suppressed by YAP^{S127A} in FNE1-YAP^{S127A} cells. Each bar represents the mean ± SEM ($n = 3$ technical replicates). Data were analyzed for significance using unpaired t test. A value of $P < 0.05$ was considered statistically significant. *$P < 0.05$, **$P < 0.01$; ***$P < 0.001$, when compared with MX control (CTRL). Exact P values for each gene: $P < 0.0001$ for *STAT3*; $P = 0.0004$ for *STAT4*; $P = 0.0448$ for *STAT5*; $P = 0.0002$ for *STAT6*; $P = 0.0027$ for *IRF9*. Source data are available online for this figure.

 © The Author(s)

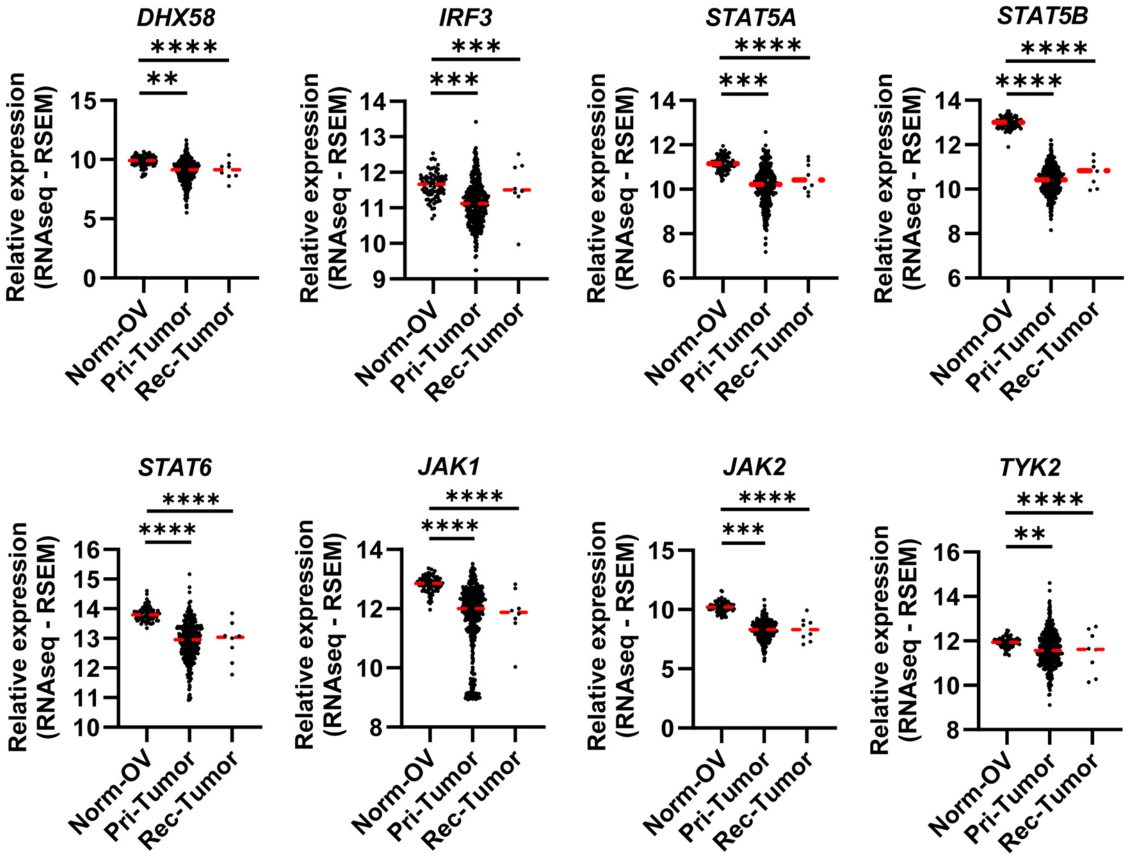

Figure EV4. Expression of genes encoding the key molecules of the innate immune signaling pathway in normal ovarian tissues, primary ovarian tumors, and recurrent ovarian tumors.

The TCGA TARGET GTEx study online tool (https://xenabrowser.net/) was used to compare the expression of genes encoding the putative HPV receptor molecules in normal ovarian tissues ($n = 88$ normal ovarian samples), primary ovarian tumor ($n = 418$ patient samples), and recurrent ovarian tumor tissues ($n = 8$ patient samples). Data were from the UCSC RNA-seq Compendium, where TCGA, TARGET, and GTEx samples are re-analyzed using the same RNA-seq pipeline. Extracted data were analyzed for significance using the one-way ANOVA followed by the Tukey's post hoc test. A value of $P < 0.05$ was considered statistically significant. ***$P < 0.001$; ****$P < 0.0001$, when compared to the normal control group (Norm-OV). Exact P values for each gene are presented with the source data of this figure, which is available online. Source data are available online for this figure.

© The Author(s)

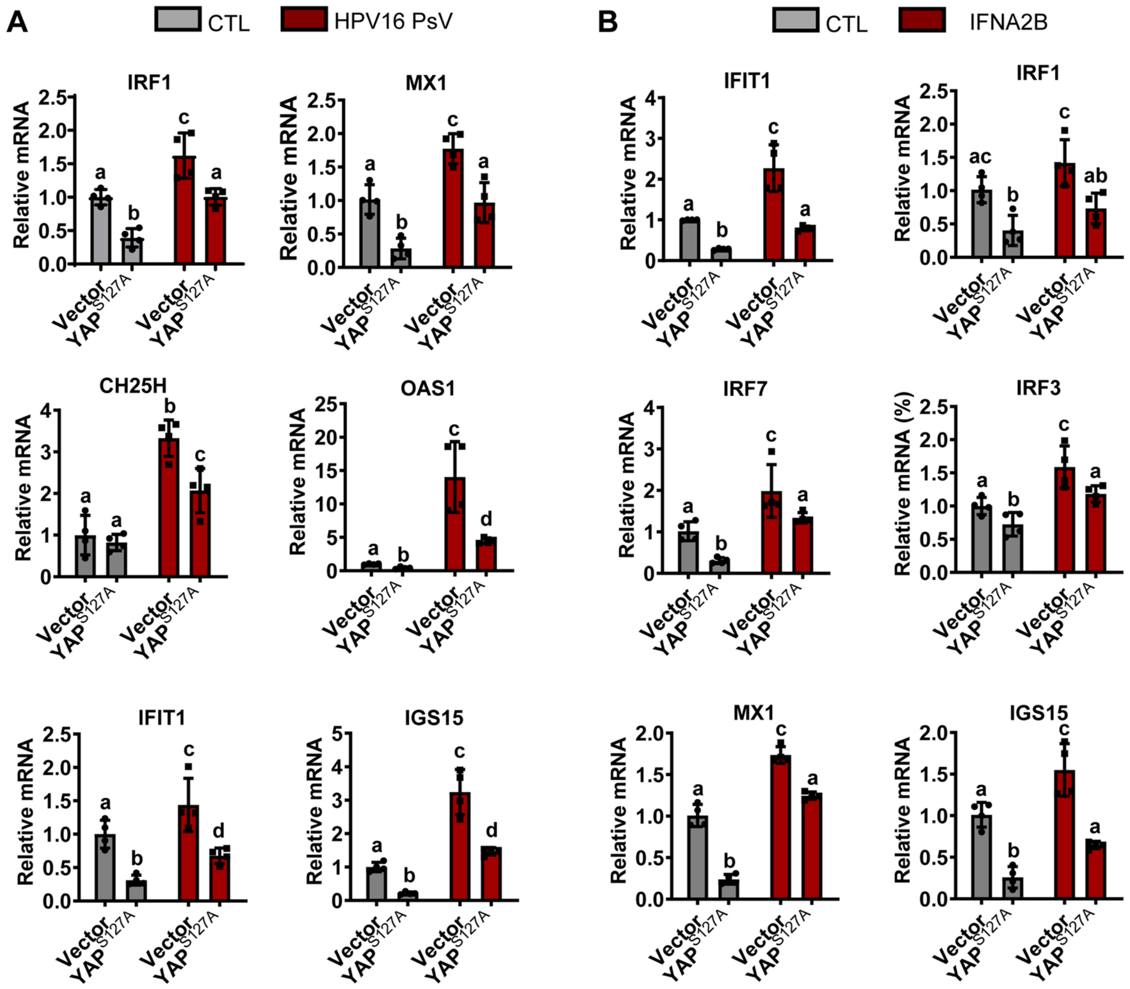

Figure EV5. Constitutive activation of YAP1 blocks basal, pathogen-induced, or IFNα2b-induced production of antiviral molecules in FTECs.

(**A**) Quantitative data showing mRNA levels of several major antiviral interferon-stimulated genes (ISGs) in control (FNE1-MX) and YAP^{S127A}-expressing FNE1 (FNE1-YAP^{S127A}) cells with or without HPV16 pseudovirions treatment. (**B**) Quantitative data showing mRNA levels of major components of the JAK/STAT/IRF9 pathway and some antiviral ISGs in FNE1-MX and FNE1-YAP^{S127A} cells with or without IFNα2b treatment. Each bar represents the mean + SEM ($n = 4$ technical replicates). Bars with different letters are significantly different from each other. Data were analyzed for significance using the two-way ANOVA followed by the Tukey's multiple comparisons post hoc test. A value of $P < 0.05$ was considered statistically significant. Exact P values between the compared groups for each gene are presented with the source data of Fig. EV5A and Fig. EV5B, which are available online. Source data are available online for this figure.

 © The Author(s)
